# Evaluations of the Peroxidative Susceptibilities of Cod Liver Oils by a ^1^H NMR Analysis Strategy: Peroxidative Resistivity of a Natural Collagenous and Biogenic Amine-Rich Fermented Product

**DOI:** 10.3390/nu12030753

**Published:** 2020-03-12

**Authors:** Benita C. Percival, Angela Wann, Richard Zbasnik, Vicki Schlegel, Mark Edgar, Jie Zhang, Gilbert Ampem, Philippe Wilson, Adam Le Gresley, Declan Naughton, Martin Grootveld

**Affiliations:** 1 Leicester School of Pharmacy, De Montfort University, The Gateway, Leicester LE1 9BH, UK; P11279990@myemail.dmu.ac.uk (B.C.P.); P10388223@365.dmu.ac.uk (A.W.); philippe.wilson@dmu.ac.uk (P.W.); 2 Department of Applied and Human Sciences, Kingston University London, Penrhyn Road, Kingston-upon-Thames KT1 2EE, UK; k1741966@kingston.ac.uk (G.A.); A.Legresley@kingston.ac.uk (A.L.G.); D.Naughton@kingston.ac.uk (D.N.); 3 Natural Product Analysis Laboratory, Department of Food Science and Technology, University of Nebraska-Lincoln, 1901 N 21st Street, Lincoln, NE 68588-6205, USA; zbasnik@unl.edu (R.Z.); vschlegel3@unl.edu (V.S.); 4 Department of Chemistry, University of Loughborough, Epinal Way, Loughborough, LE11 3TU, UK; M.Edgar@lboro.ac.uk; 5 Green Pasture Products, 416 E. Fremont Street, O’Neill, NE 68763, USA; gpplab@greenpasture.org

**Keywords:** cod liver oil, fermented foods, peroxidative susceptibility/resistivity, lipid oxidation products, aldehyde toxins, ^1^H NMR analysis, biogenic amines, antioxidants, flavanones, collagen-derived antioxidants, NMR-based Thermo-Oxidation Resistivity Assay (TORA)

## Abstract

High-resolution ^1^H nuclear magnetic resonance (NMR) analysis was employed to molecularly screen the lipid, lipid oxidation product (LOP), and antioxidant compositions of four natural (unrefined) cod liver oil (CLO) products. Products 1–3 were non-fermented CLOs, whilst Product 4 was isolated from pre-fermented cod livers. Supporting analytical data that were acquired included biogenic amine, flavanone, tannin, phenolic antioxidant, α-tocopherol, and oxygen radical absorbance capacity (ORAC) determinations by recommended HPLC, LC/MS/MS, or spectrophotometric methods. SDS-PAGE, HPLC, and ^1^H NMR analyses investigated and determined collagenous antioxidants and their molecular mass ranges. ^1^H NMR analysis of aldehydic LOPs was employed to explore the susceptibilities/resistivities of each CLO product to peroxidation that is induced by thermal stressing episodes (TSEs) at 180°C, or following prolonged (42 day) storage episodes at 4 and 23 °C. Product 4 displayed extremely high ORAC values, which were much greater than those of Products 1–3, and that were predominantly ascribable to significant levels of peroxidation-blocking and/or aldehyde-consuming collagenous polypeptides/peptides and ammoniacal agents therein. Significantly lower levels of toxic aldehydes were generated in the pre-fermented Product 4 during exposure to TSEs, or the above long-term storage episodes. These results confirmed the enhanced peroxidative resistivity of a fermented, antioxidant-fortified natural CLO product over those of non-fermented unrefined products. Product 4: Green Pasture Blue Ice™ Fermented Cod Liver Oil.

## 1. Introduction

The global fish (marine) oil market is forecast to reach $3.69 billion by 2025, according to a recent report by Allied Market Research, entitled ‘Global Fish Oil Market (Application, Species and Geography)—Country Analysis, Size, Share, Trends, Company Profiles, Demand, Growth, Opportunities, and Forecast, 2015–2025’ [1]. Fish oil is extensively employed in numerous human and animal nutrition applications, both as foods and food supplements, in view its high content of essential long-chain, ‘health-friendly’ polyunsaturated omega-3 (n-3) fatty acids (O-3 FAs). This market is experiencing a major boost in view of the popularity of O-3 FA-rich fish oils in various applications, such as supplements and functional foods (nutraceuticals), as well as a wealth of pharmaceutical applications. Nutraceuticals are defined as ‘a food or part of a food that allegedly provides medicinal or health benefits, including the prevention and treatment of disease’ [2], and therefore a nutraceutical might represent a naturally nutrient-rich or medicinally-active food source or component, with the latter classification including O-3 FA-rich fish oils. Recent reviews of the potential health benefits that are offered by compounds classified as nutraceuticals, and the status of current research focused on this area, are available in [3,4,5].

In addition to essential O-3 FAs, such as docosahexaenoic acid (DHA) and eicosapentaenoic acid (EPA), major bioactive agents that are found in such marine oils are vitamins A (retinol) and D3 (cholecalciferol), and cod liver oil (CLO) represents a rich dietary source of these essential nutrients. Significant levels of tocopherols (vitamin E and its isomers) are also present. When employed, harsh refinement processes for these products unfortunately remove the majority of natural retinol and cholecalciferol present in CLOs and, therefore, these are added as synthetic derivatives to refined products, the former usually as its retinyl palmitate ester derivative [6].

However, recent developments have focused on an alternative perspective involving the now increasingly documented beneficial health prospects that are offered by more polar phospholipids (PLs) from both marine and animal sources, which have been shown to exert highly promising anti-inflammatory effects that a variety of positive bioactivities mediates [7].

The multicomponent analytical ability of high-resolution nuclear magnetic resonance (NMR) spectroscopy allows for the rapid, virtually non-invasive, non-destructive, and simultaneous study of complex mixtures of agents that are present in edible oil products, and more generally for determining the nutrient status of foods [8,9,10]. Further advantages offered are that the technique has no major requirements for detailed knowledge of sample composition prior to analysis (i.e. it is an untargeted technique), and chemical shifts, coupling patterns, and coupling constants of resonances that are contained within ^1^H and ^13^C NMR spectra of such samples provide a very high level of confirmatory evidence for the molecular structures of a multitude of lipidic and lipid-soluble agents detectable. 

NMR techniques are also of significant use for addressing and determining the acylglycerol chain FA profiles and unsaturation status of such oils [6,11,12] and, in their ^13^C form, for the detection and estimation of free [13] and *trans*-FAs [6]. Moreover, they offer a very high level of analytical information on lipid-soluble agents that are present at much lower concentrations than those of acylglycerols, e.g. di- and monoacylglycerols, antioxidants, such as α-tocopherol (α-TOH) and ‘health-friendly’ phytosterols, etc. [14,15,16,17]. Of particular value is their ability to detect and determine a very wide range of lipid oxidation products (LOPs), either in unheated oils, or those exposed to high-temperature frying episodes [15,18,19]. Indeed, this technique can monitor a variety of secondary aldehydic and epoxy-acid LOPs, along with their conjugated hydroperoxydiene (CHPD) or hydroperoxymonoene (HPM) precursors. Even low-field (60 MHz operating frequency) benchtop ^1^H NMR analysis has also been proven to offer a similar sensitivity to, and much improved selectivity than that of Fourier-transform infra-red (FTIR) analysis and, hence, serves as a superior screening tool for the simultaneous analysis of a range of lipids that are present in culinary oil samples [20]. NMR fingerprinting analysis has served as a valuable analytical strategy for the detection of edible oil adulteration, and the authentication of their geographical sources [17]. Moreover, ^1^H and ^13^C NMR strategies have both seen significant application in the characterisation and authentication of marine oils. For example, the ^13^C approach can readily determine the glycerol backbone substitutional status of essential DHA and EPA in marine oils [6]. However, to date, studies reporting the ability of NMR screening techniques to probe the antioxidant status and peroxidative susceptibilities of fish oil products remain limited, e.g. [6,21].

The fermentation of fish food sources with lactate-generating and salt-tolerant bacterial forms generates a rich plethora of valuable products, for example, a range of biomolecules with protective microbicidal and/or antioxidant functions. Indeed, glycogen is degraded to provide lactate and many other carboxylic acid anions, such as acetate and propionate, triacylglycerols are hydrolysed to free FAs and selected flavour and aroma compounds (albeit to a limited extent), and proteins are consumed to yield smaller polypeptides, peptides and free amino acids, biogenic amines, and volatile nitrogen compounds (including ammonia), along with further odorous and flavour-enhancing agents [22]. 

Hur et. al. have thoroughly reviewed recent developments in the fermented food research area [23]. Although the antioxidant activities of fermented foods have previously been screened by their oxygen radical absorbance capacity (ORAC) and/or their 1,1-diphenyl-2-picryl hydrazyl (DPPH) and 2,2′-azino-*bis*(3-ethylbenzo-thiazoline-6-sulfonic acid) (ABTS) radical scavenging activities, their total phenolic content (TPC) values, and their overall reducing powers [24], to date, the multicomponent analytical ability of high-resolution NMR analysis has not been exploited for this purpose.

Therefore, we have employed high-resolution (predominantly 600 MHz) ^1^H NMR spectroscopy, coupled with a wide range of additional analytical strategies, in this study in order to fully evaluate the molecular compositions of four commercially-available, natural (unrefined) cod liver oil (CLO) products. These studies included determinations of the molecular nature and concentrations of a wide range of antioxidant species to compare the results acquired for a CLO product (Product 4) generated from pre-fermented cod livers (henceforth termed ‘fermented CLO’) with those of three non-fermented ones (Products 1–3). Products 1–3 were selected for comparative evaluations on the basis that they were unrefined natural products but were not generated from fermented cod liver matrices.

One further objective was to determine the susceptibilities/resistivities of UFAs in these products to/against peroxidation to explore the protective capacities of their antioxidant compositions against this autocatalytic chain reaction process. For this purpose, these CLOs were exposed to thermal stressing episodes (TSEs, i.e. 0–90 min. at 180 °C), and a range of secondary aldehydic LOPs were determined in samples collected at increasing time-points by high-field ^1^H NMR analysis. We have termed this ^1^H NMR approach as the NMR-Based *Thermo-Oxidation Resistivity Assay* (acronym TORA), and this serves as a viable and highly valuable alternative to the high temperature-based accelerated Rancimat® method that is available for determining the peroxidative resistivities of edible oils [25], and also offers a substantially higher level of selectivity. The CLO aldehydic LOP levels were also monitored prior and subsequent to prolonged (42-day) temperature-dependent storage episodes (TDSEs) at both 4 and 23 °C. The differences observed between the nature and levels of aldehydic LOPs generated during the exposure of CLO products to such TSEs and TDSEs are discussed in the context of their antioxidant and FA compositions. 

Rationale for the Study:

- to determine whether high-resolution ^1^H NMR analysis can be successfully applied to determine and monitor the FA composition, antioxidant levels, and chain-breaking antioxidant status of CLO products, and the ability of this technique to provide valuable supporting information to that obtained from established, more conventional analytical methods that are available for these purposes;
- to investigate whether the pre-fermentation of cod liver sources successfully fortifies the antioxidant composition of CLO products arising therefrom, and the molecular basis of this fortification; and,
- to apply the newly-developed NMR-based TORA TSE and TDSE approaches to determine the relative abilities of CLO product unsaturated fatty acids (UFAs) to resist peroxidation, and determine whether antioxidant-fortifying, pre-fermentation of their cod liver sources renders them more resistant to such oxidation.

## 2. Materials and Methods

### 2.1. CLO Samples

CLO Products 1–4 were purchased from US retail stores (Product 4 was a fermented natural product that was manufactured by Green Pastures LLC, 416 E. Fremont O’Neill, NE 68763, USA, and Products 1–3 were competing non-fermented natural (unrefined) US products). A total of 4–6 separate batches of these products were subjected to each type of non-NMR chemical/biochemical analysis described below.

The sampling strategy that was employed for different batches of Products 1–3 involved their random purchase from separate, independent US retail outlets throughout the US state of Nebraska. The serial numbers of each of these was checked to ensure that they arose from different manufacturing batches. Similarly, differing separate batches of Product 4 were randomly selected by independent visitors to its manufacturing site throughout a 6-month period.

Fermented CLO (Product 4) was prepared from the fermentation of Pacific cod livers. These livers were frozen (−20 °C) within 40 min. following their harvest from the Pacific Ocean, and then transported to a preparation facility whilst frozen. Fermented CLO was produced from these cod liver sources while using a novel and propriety fermentation technology. Briefly, cod livers were loaded into the fermentation tank, and both salt and the fermentation starter agent were added to initiate the process. The tank was completely sealed during the fermentation and, following periods of between 28–84 days, the raw CLO accumulated, and was then removed from the tank. Following fermentation, the raw CLO was then centrifuged, filtered to remove particulate matter, and subsequently packed.

On arrival, each CLO product was de-identified in the laboratory via its transference to coded but unlabelled storage containers. The samples were then stored in a darkened freezer at −80 °C until ready for analysis, usually within 24 h of their arrival. The molar % contents of DHA, EPA, total UFAs, and saturated fatty acids (SFAs) of these oils were determined by modifications of the ^1^H NMR methods that were developed in [26] and [27], and that of total O-3 FAs in these samples was estimated by the previously reported ^1^H NMR method [6,26].

Section S1 of the Supplementary Materials provides details regarding the FA composition of CLO products, which were analysed by both ^1^H NMR and gas chromatographic (GC) analysis approaches (the latter involving the AOAC 996.06 method). Table S1 lists the mean±SEM ^1^H NMR-determined molar % contents of total SFAs, UFAs, and O-3 FAs, and those of DHA and EPA, in Products 1–4. Briefly, the DHA and EPA contents of these products ranged from 9.3–12.3 and 9.7–15.5 molar %, respectively, with Product 4 having the lowest and highest levels, respectively, of these FAs. The results arising from the GC analysis of Product 4 are also available in this section, as are details of the AOAC method employed for this purpose.

From these results, our ^1^H NMR analyses of the FA profiles of Product 4 were in very good agreement with these GC-determined values. Similarly, the *trans*-FA values that were determined by this GC method were in excellent agreement with those determined by our previously reported ^13^C NMR analysis strategy [6], i.e. 1.72–2.18 molar %. 

### 2.2. Authentic Reference Aldehydic LOPs

Authentic aldehydes employed for ^1^H NMR reference purposes, e.g. *n*-hexanal, *n*-octanal, *trans*-2-octenal, *trans*-2-nonenal *trans, trans*-deca-2,4-dienal, etc., were purchased from the Sigma–Aldrich Chemical Co. (Gillingham, UK). 

### 2.3. Exposure of CLO Samples to TORA Thermal-stressing and Temperature-dependent Storage Episodes (TSEs and TDSEs Respectively), and Their Preparation for ^1^H NMR Analysis 

All of the CLOs were heated at a temperature of 180 °C for periods of up to 90 min. according to our specified TSEs, i.e. a minor modification of that previously described in [26]. Section S2.1 provides full details regarding these TSE experiments, TSE sampling time-points (0–90 min.), and the preparation of the samples collected therefrom for ^1^H NMR analysis (Supplementary Materials).

Each CLO product was also exposed to long-term (42-day) TDSEs in the dark at both refrigerator (4 °C) and ambient temperatures (23 °C). For this purpose, the products were stored in light-excluding containers in a refrigerator section, which was continually unexposed to light (4 °C), or a darkened laboratory container at a mean ambient temperature of 23 ± 1 °C (mean ± SD). On day 42, the samples were collected and then immediately prepared for ^1^H NMR analysis, as described in Section 2.4. This analysis was completed within 2 h of sample collection, and n = 3 replicate samples were analysed in each case.

### 2.4. Preparation of ^2^H_2_O and C ^2^H_3_O^2^H Extracts of CLO Products 1–4

Section S2.2 provides full details regarding the preparation of these deuterated solvent extracts for ^1^H NMR analysis (Supplementary Materials).

### 2.5. H NMR Analysis

Section S2.3 of the Supplementary Materials outlines information regarding further sample preparation measures taken for ^1^H NMR analysis, NMR spectrometer facilities employed and their operating frequencies, reference calibration standards and typical pulsing conditions used, etc. Details concerning the acquisition of 2D correlation spectroscopy (COSY) and total correlation spectroscopy (TOCSY) [14,15,18], and Carr-Purcell-Meiboom-Gill (CPMG) [28,29] spectra, are also available in this section. 

Full details regarding the preprocessing of ^1^H NMR profiles, the determinations of different aldehydic LOP classification, and their lower limits of detection and quantification (LLOD and LLOQ, respectively) are available in Section S2.4 of the Supplementary Materials.

### 2.6. Experimental Design and Statistical Analysis

For experiments featuring the exposure of CLO products to TORA TSEs, the experimental design for univariate analysis of the total acylglycerol content-normalised ^1^H NMR aldehyde classification intelligently-selected bucket (ISB) intensity datasets involved an analysis-of covariance (ANCOVA) model, which incorporated two prime factors and a total of four sources of variation: (1) that ’between-CLO products’, qualitative fixed effect (O*i*); (2) that ‘between-sampling time-points’ quantitative fixed effect ’nested’ within ’CLOs’ (T*_(i)j_*); (3) the CLO product x time-point first-order interaction effect (OT*_ij_*); and, (4) the ‘between-replicate’ random effect nested within factors (1) and (2). Equation 1 shows the mathematical model for this experimental design, in which y*_ijkl_* represents the (univariate) aldehyde ISB dependent variable values observed, μ its overall population mean value in the absence of any significant, influential sources of variation, and e*_ijkl_* the unexplained error (residual) contribution.

*y_ijkl_* = μ + O*_i_* + T*_(i)__j_* + OT*_ij_* + R*_(ij)k_* + e*_ijkl_* (1)

ANCOVA was conducted with *XLSTAT2016* software (Addinsoft, Paris, France). *Post-hoc* analysis of significant differences that were observed between individual CLO products and sampling time-points were performed while using Tukey’s test.

Further analysis of the univariate aldehydic LOP level data was performed by comparisons of their least square mean (LSM) values. For this work, adjusting for the major ‘between-heating time-point’ source of variation was utilized to compute LSM values for the ‘between-CLO product’ factor, and vice-versa for the latter. The statistical significance of these LSM differences were also determined by Tukey’s *post-hoc* test. 

Principal component analysis (PCA) and Pearson (linear) correlation analysis of the CLO NMR-based aldehyde concentration dataset were performed using the *Metaboanalyst 4.0* software module option. The datasets (mmol. aldehyde/mol. FA) were analysed following cube root-transformation and Pareto scaling. Heatmap and correlation feature diagrams were also generated with this software module. ROC curve analysis was also performed using *Metaboanalyst 4.0*. For this purpose, a support vector machine (SVM) model building strategy was explored with 2–9 variables. The ROC curves were developed via Monte Carlo cross-validation involving a balanced sub-sampling process (cubed root-transformed and Pareto-scaled total acylglycerol-normalised aldehyde levels).

### 2.7. Analysis of Antioxidants (including Tocopherols), Phenolics, Flavonoids and Flavonones, Anthocyanins, Tannins, Chlorophylls, Carotenes, and Retinol in CLOs

Full details, materials employed, and extraction strategies for the analysis of total phenolics, flavonoids and flavanones, anthocyanins, and tannins are available in Sections S3–S10 of the Supplementary Materials, as are the methods employed for the determination of α- and γ-tocopherols and retinol. Carotenoids (β-Carotene and total) and chlorophylls were determined, as described in Section S11.

### 2.8. Determination of the Oxygen Radical Absorbance Capacities (ORACs) of CLO Products

The ORAC values of each CLO product evaluated were determined by an adaption of the method of Huang et. al. [30] (Supplementary Materials Section S9). This microplate fluorescence reader-based evaluation system is particularly relevant to this study, since it is based on the ability of minor components, antioxidant or otherwise, contained in the oil products that were tested to scavenge peroxyl radicals. ORAC values were expressed in mmol/kg units. Full details of this analytical method are available in Section S12 of the Supplementary Materials. The statistical significance of differences between the mean ORAC values of n = 5 samples of separate batches of Product 4, and those of four different non-fermented natural CLO batches were determined using a two-sample t-test performed on natural logarithmically (ln)-transformed data; the ln transformation successfully homogenized differing intra-sample variances (*p* = 0.11, F variance ratio statistic).

### 2.9. Biogenic Amine, Total Protein, Collagen, Ammonia and Water (moisture) Analyses

Biogenic amine analysis was conducted by the LC/MS/MS technique (details are available in Section S13 of the Supplementary Materials). Full outlines of the standard calibration methods employed for the analysis of total protein, collagen, and ammonia in Product 4 are available in Section S14 of the Supplementary Materials, and that for its moisture content is described inSection S15.

Ethical Approval: This article does not contain any investigations with human participants or experimental animals performed by any of the authors.

## 3. Results

### 3.1. ORAC Values and Antioxidant Status of CLO Products

ORAC determinations were first performed in order to explore the peroxyl radical-scavenging antioxidant capacity of each CLO product. These measurements revealed that Product 4 had ‘between-batch’ mean ± SEM values of 91.4 ± 19.5 mmol. trolox equivalents/kg (*n* = 5 samples, each from separate batches), whereas those of *n* = 5 different batches of the non-fermented natural Products 1, 2, and 3 were found to be 4.9 ± 1.0, 46.2 ± 4.6, and 5.9 ± 1.1 mmol. trolox equivalents/kg, respectively. The literature ORAC value data available for antioxidant-rich virgin olive oil range from only 1.8–6.2 mmol. trolox equivalents/kg (with positively-correlating total phenolic levels of 50–254 mg/kg), whereas those for the refined olive and peanut oil products were only 1.0–1.6 mmol. trolox equivalents/kg (phenolic contents only 1–10 mg/kg) [31]. These data provided a high level of evidence for Product 4’s powerful peroxidative resistance. However, it should also be noted that one of the other products tested (Product 2) contained added rosemary extract and natural mixed tocopherols, and these additions may account for its relatively high mean ORAC value. However, Product 3 also contained an unspecified concentration of cholesterol, along with natural levels of vitamins A and E. Similarly, Product 1 was a cold-pressed virgin CLO product, which was supplemented with small amounts of added rosemary extract and natural vitamin E. The substantial difference that was observed between the much elevated mean ORAC value of Product 4 and that of the other products combined was found to be very highly statistically significant (*p* = 7.53 x 10^-5^, two-sample t-test). Principally, Product 3 contained alpha-tocopherol (α-TOH) added as its acetate ester, which fails to offer UFAs protection against peroxidative damage unless α-TOH itself is liberated therefrom via any hydrolysis reactions occurring. 

Furthermore, a range of antioxidant and potential antioxidant species, including flavonoids, flavonones, total phenolics, tocopherols, carotenoids, tannins, anthocyanins, and retinol, was determined in four or five separate batches each of Products 1–4. Table 1 shows these results.

The total flavonoids, flavanones, phenolics, tannins, and anthocyanins were undetectable, i.e. below the limits of quantification, in Products 1–3 (Table 1); however, Product 4 was found to have substantive contents of flavanones, phenolics, and tannins. Although all tested products had similar concentrations of chlorophyll A, that of chlorophyll B was much greater in Product 4. Moreover, the total carotenoid contents of Products 1–3 were only ca. 40% of that present in Product 4 (*p* < 0.01). Therefore, higher levels of these antioxidants and chlorophyll B observed in Product 4 appear to arise from the fermentation process that was employed during its commercial production. 

However, no significant differences were found between the mean α-TOH contents of each of the four CLOs. Moreover, for retinol, the only significant difference found was that between Products 2 and 3, with the latter being almost two-fold higher. One further noteworthy observation is that the ‘between-batch’ variation of some of the analytes monitored in Table 1 was higher for Product 4 than it was for those of Products 1–3. For example, the intra-product variance for the Product 4 α-TOH concentrations determined was significantly greater than those of Products 1–3 (*p* < 10^-3^, Bartlett’s test), but this was not the case for the retinol levels. This wider variance in Product 4 levels observed for some of these analytes might also be attributable to its fermented route of production.

The results acquired confirmed that although Product 4 contained peroxidatively-significant, near-millimolar concentrations of at least some of these agents (predominantly tyramine and 2-phenylethylamine), such amines were completely undetectable in all analysed batches of Products 1–3, as expected (Table 2).

Further experiments conducted involved the monitoring of biogenic amines in Product 4 samples both before and after exposure to a 90 min. heating episode at 180 °C, according to our TSE protocol (Section 2.3). The data acquired clearly demonstrated the complete removal of these agents from this CLO product when heated in this manner.

Section S16 of the Supplementary Materials provides full details of the results arising from these experiments, together with possible explanations for them. 

### 3.2. Investigation of an Unusual Broad ^1^H NMR Resonance in ^1^H NMR Spectral Profiles of a CLO Product: Assignment to Collagen/Collagen Hydrolysate Products and Ammonia

**Figure 1:**
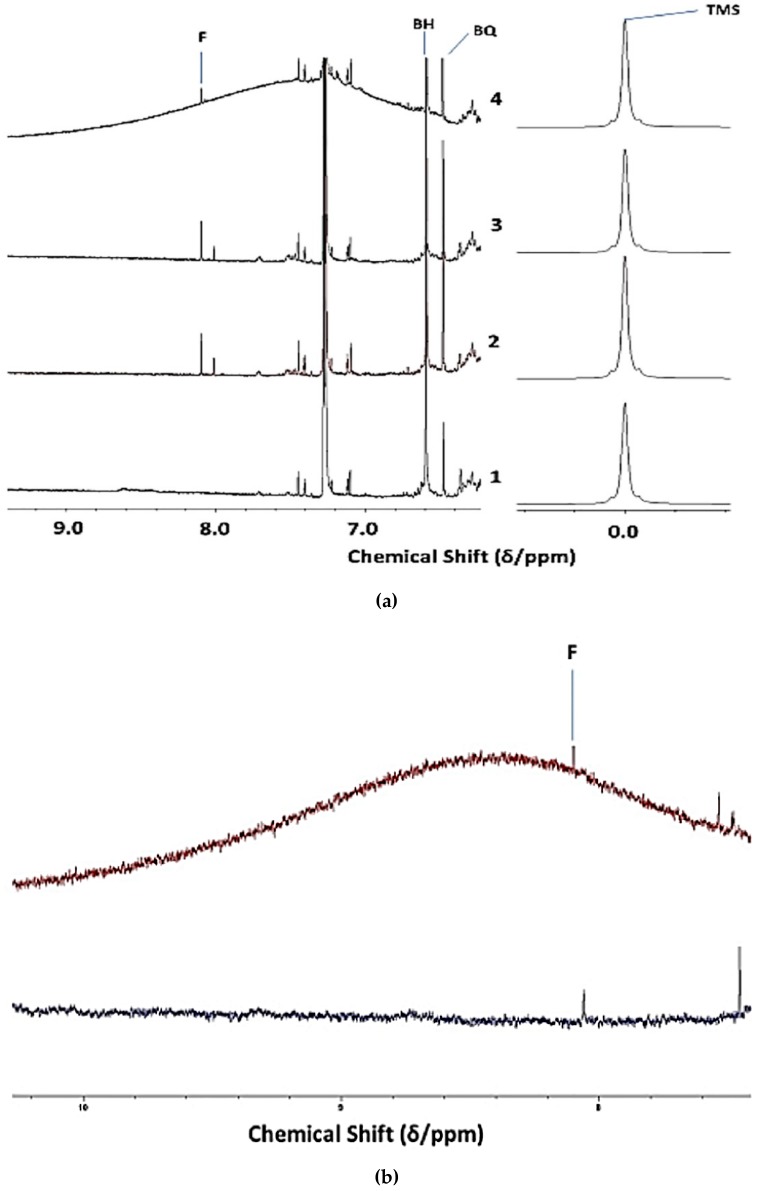

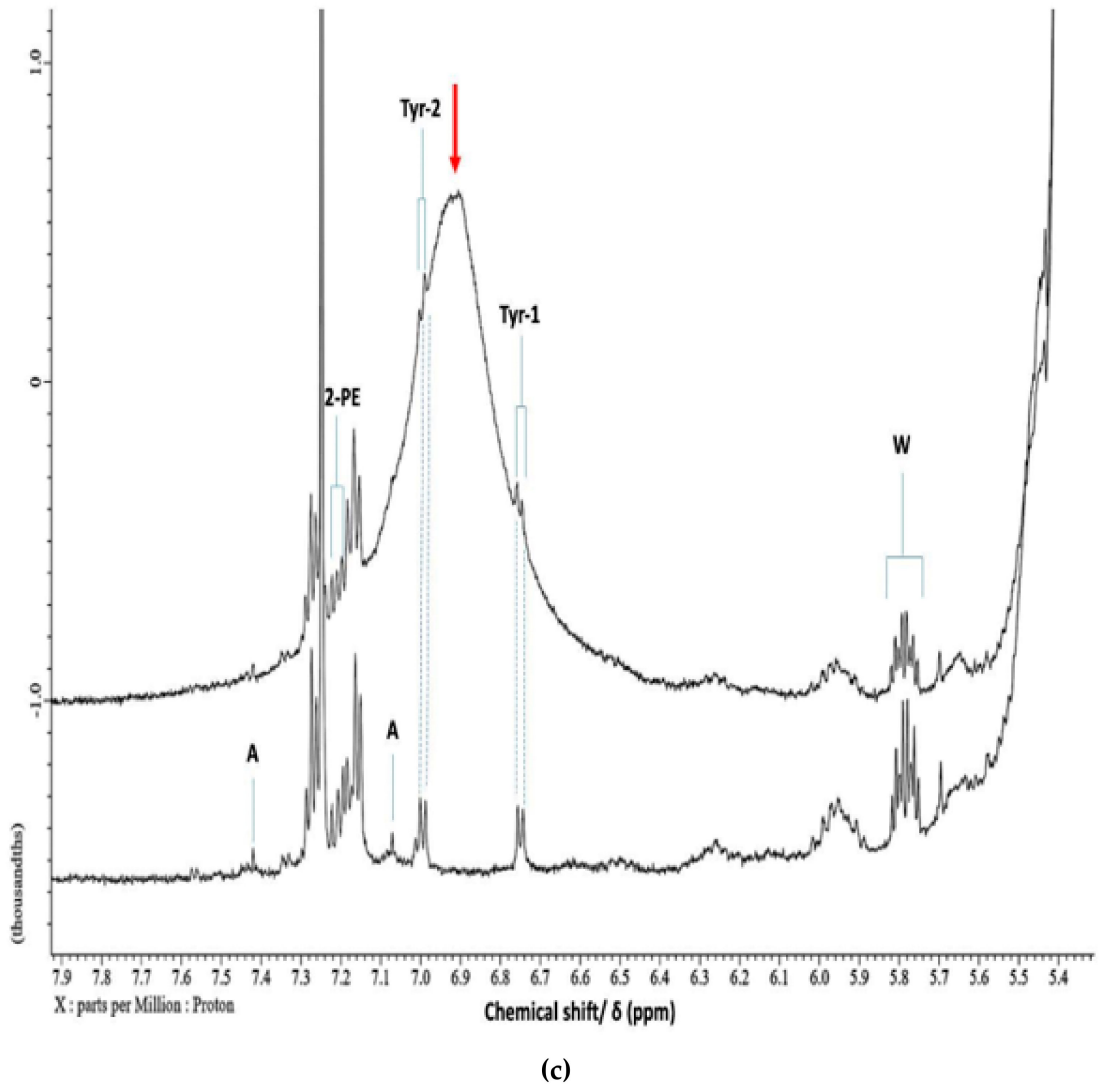
**^1^H NMR Detection of Collagenous Agents in Fermented CLO Product 4.**^1^H NMR detection of collagenous peptides/polypeptides in fermented CLO product. (**a**) Expanded 6.20–9.40 ppm region of 500 MHz ^1^H NMR profiles acquired on Products 1–4, showing the unusual, very broad resonance detectable in that of Product 4 only; (**b**) Expanded 8.40–10.30 ppm region of the 500 MHz ^1^H NMR spectrum of Product 4 in C^2^HCl_3_ solution acquired prior (top, red) and subsequent to (bottom, blue) micro-extraction with ^2^H_2_O (bottom). (**c**) Expanded 5.30–7.90 ppm region of the 600 MHz ^1^H NMR spectrum of Product 4 in C^2^HCl_3_ solution before (top) and after (bottom) treatment with a 10 µL volume of ^2^H_2_O (bottom). The right-hand side panel of (**a**) shows partial spectra containing the tetramethylsilance (TMS) internal chemical shift reference resonance. Abbreviations: 2-PE, 2-phenylethylamine aromatic proton resonances (centered at δ = 7.23 ppm) [31]; Tyr, tyramine aromatic ring proton resonances; A, residual CHCl_3_^13^C satellites (*J* = 209 Hz); BH, di-*tertiary*-butyl-hydroquinone (DTBHQ) 3,6-position aromatic proton singlet resonance; BQ, 3,6-position aromatic proton singlet of 2,5-ditertiary-butyl-benzoquinone.; F, formic acid HCO_2_H; W, multiplet (δ = 5.74-5.86 ppm) of the -CH=CH_2_ proton of unsaturated *ω*-1 FAs.

Evidence for the identities of agents that are responsible for the high ORAC values and, hence, peroxidative resistivity of Product 4 was provided by high-field ^1^H NMR analysis experiments (600 MHz). Indeed, an examination of the 6.40–10.00 ppm regions of spectra acquired on *n* = 6 unheated batches revealed a very broad resonance centered at ca. *δ* = 6.40 to 9.48 ppm (highly variable maximal intensity, centralised chemical shift values of 6.87, 7.40, 7.80, 7.95, 8.43, and 9.18 ppm were typically observed), and that generally spanned chemical shift ranges of ≥ 2.5 ppm (Δ*v*_1/2_ ≥ 250 Hz) This unusual signal was not at all present in corresponding spectra of Products 1-3 (Figure 1a). Micro-extraction of the sample with the ^1^H NMR profile shown in this spectrum with ^2^H_2_O removed this resonance from the ^1^H NMR profiles (Figure 1b), an observation that might be consistent with the greater solubility of the agent(s) giving rise to it in aqueous systems. Moreover, following an equilibration period of 1.0 h at ambient temperature, this signal was also completely removed from the spectra that were acquired following its treatment with a very small C^2^HCl_3_-miscible volume of ^2^H_2_O (Figure 1c). This signal was also removed with a further 1:5 (v/v) dilution of analysis solutions with C^2^HCl_3_ (the red arrow in Figure 1c indicates this very broad ^2^H_2_O-extinguishable resonance). Additional experiments involved monitoring the intensity of this broad resonance as a function of TSE heating time at 180 °C (Section 2.3), and the ^1^H NMR data acquired demonstrated that it was eliminated from spectra with increasing sampling time-point, a substantial decrease in its intensity occurring within a 10–20 min. heating duration. Full details of these investigations will be reported in a second, follow-up publication in view of this observation’s novelty, and also the expansive number of further experiments performed to fully establish the identity/identities of this broad Product 4 resonance. However, in summary, the results from these studies revealed that this ^1^H NMR resonance is predominantly ascribable to an admixture of protein, predominantly collagen and collagen hydrolysate peptide linkage-CO-NH- protons, together with lower levels of the fermentation product ammonia, the latter as C^2^HCl_3_-solubilised ammonium ions. Broad amide-/peptide-CO-NH- function resonances have highly variable chemical shift values (δ = 5–9 ppm) [32], and these are further complicated by their macromolecular nature when present in proteins/polypeptides.

The high frequency chemical shift values that are associated with this deuterium-exchangeable resonance precludes assignments to aliphatic alcohol-OH, amine-NH_2_, and carboxylic acid-COOH functions (limited to the δ = 0.5–5.0, 0.5-4.5, and 10.0–13.2 ppm ranges, respectively), although it is within the range for the phenolic-OH group ^1^H NMR signals (δ = 4–8 ppm) [32].

Intact collagen, along with the higher molecular mass fractions of its hydrolytic degradation products, have very short T_2_ values under our experimental conditions due to their high molecular masses (up to 300 kDa for intact collagen). However, this is also the case for ammonia’s ^1^H nuclei in view of chemical exchange phenomena and associated ^14^N nucleus couplings. Indeed, the application of the macromolecule-filtering CPMG pulse sequence to unheated Product 4 solutions in this solvent gave rise to the complete removal of this signal from the ^1^H NMR profiles that were acquired. Further resonances that were removed from these spectra with this pulse sequence were a series of much sharper α-CH amino acid proton resonances that were located within the 4.2–4.7 ppm range [33,34], which presumably also arise from collagenous sources, e.g. its lower molecular mass hydrolytic degradation products. However, this resonance-dampening effect of the CPMG pulse sequence was also observed on a very broad resonance (centered at δ= 9.2 ppm, but spanning the entire 7.1–12.0 ppm range) that was found in the spectra of C^2^HCl_3_ sequentially treated with (1) NH_3_ and (2) glacial acetic acid to generate NH_4_^+^ therefrom (data not shown). 

The total protein, collagen, and ammonia contents of a number of different batches of this fermented CLO product were found to be up to 1.6% (w/w), 1.5% (w/w), and 80 mg/kg, with the latter presumably largely present as ammonium ions, possibly as C^2^HCl_3_-soluble ion-pair complexes with free FA carboxylate functions. Indeed, Product 4 has higher concentrations of free FAs in view of the fermentation process that is involved in its commercial preparation [22]. Water, which might aid the solubility, emulsification, and/or dispersion of collagenous degradation products in Product 4, was found to be present at levels ranging from 0.3 to 1.0 % (w/w) (mean content *ca.* 0.5% (w/w)). The total collagen concentrations were determined as free hydroxyproline in hydrolysates of CLO sample extracts, using an HPLC method, as described in Section S14.2.2 of the Supplementary Materials. The total protein content of Products 1–3 was reported as none detectable, i.e. below the specified reporting limit of 0.10% (w/w).

The electronic integration of this broad signal gave an estimated single proton-equivalent concentration as high as 0.44 mol./kg. However, this level was 0.11 mol./kg. for ammonium ion with 4 ^1^H NMR-equivalent proton contributors. These values, which are much higher than those of phenols, flavanones, tannins, and biogenic amines determined in Product 4, are, however, more similar to its estimated ORAC values. Notwithstanding, the above estimated ammonia concentration is markedly greater than that determined by the non-NMR method (*ca.* only 5 mmol./kg) and, therefore, it appears that the broad signal observed is predominantly attributable to collagenous sources, notably a variable molecular mass range of its hydrolytic degradation products, along with smaller amounts of the intact or virtually intact protein.

The collagen content of Product 4 was also investigated by a SDS-PAGE gel electrophoresis analytical strategy, and the results from these experiments revealed that it had a highly variable molecular mass range, i.e. from 5 to 270 kDa, an observation that was consistent with a high proportion of it being in the form of hydrolytic fragmentation products. Such gelatin-type hydrolysis products presumably arise from the fermentation of collagen substrates during its production. Linear regression analysis confirmed that there was a very strong positive linear relationship between the total FA-normalised overall intensity of this broad resonance and the (% w/w) protein content of this fermented CLO product (*r* = 0.99, *p* = 1.45 × 10^-4^; *n* = 5).

Similarly, there was also a strong positive correlation between Product 4’s ORAC value and its protein contents (*r* = 0.91, *p* = 0.031; *n* = 5). No significant positive correlations between ORAC values and total phenolics, flavonones, α-TOH and tannins were found. However, there were between ORAC values and the biogenic amine tyramine (*r* = 0.90, *p* = 3.56 × 10^-2^); this observation is consistent with its fermentational source. Moreover, the correlation observed between Product 4 ORAC values and 2-phenylethylamine concentrations was close to statistical significance (*p* = 0.073). However, that between this amine and tyramine was statistically significant (*r* = 0.92, *p* = 2.66 × 10^-2^), as might be expected from any between-batch differences in fermentation length or conditions during product preparation.

Although the full identity/identities of this broad Product 4 resonance remains complex and is undoubtedly multicomponent, it might be concluded that it is very unlikely to arise from phenols in view of their relatively low concentration. A further possibility is that it partially arises from phenolic-OH function-containing flavonones and/or polymeric tannins. Notwithstanding, although the final added level of the DTBHQ antioxidant was 6.58 mmol./kg (equivalent to 13.16 mmol./kg phenolic-OH group equivalents) in CLO/C^2^HCl_3_ analyte samples, this agent is clearly not also contributing towards this very broad signal, since equivalent concentrations were added to all of the CLO samples investigated here, and it was also not visible in the spectra acquired on Products 1–3. Moreover, this final laboratory-added DTBHQ level is similar to or higher than that of the combined phenol, flavanone, tannin, and tocopherol levels found in Product 4 (Table 1).

Product 4 spectra also contained relatively low intensity resonances that are ascribable to C^2^HCl_3_- and lipid-soluble low-molecular-mass aromatic compounds located within the δ = 6.70–7.36 ppm range, which are much more ^1^H NMR-observable following the removal of the broad, predominantly collagenous peptide-CO-NH- signal; these are attributable to biogenic amine antioxidants. Indeed, the multiplet resonances centered at δ = 7.23 ppm are assignable to the combined aromatic protons of 2-phenylethylamine in this solvent [35], which are present in this product at levels that are readily ^1^H NMR-detectable (up to 0.85 mmol./kg, Table 2). Moreover, two clear *p*-substituted aromatic ring doublet signals that are located at δ = 6.74 and 6.99 ppm are assignable to tyramine. These assignments were confirmed via the acquisition of ^1^H NMR spectra on the aqueous (^2^H_2_O) and C^2^H_3_O^2^H extracts of this product (Supplementary Materials Section S17 and Figure S1) [36,37]. 

Interestingly, the spectra acquired also demonstrated that singlet resonances ascribable to both DTBHQ and its corresponding benzoquinone oxidation product were also clearly visible, an example of which is shown in Figure 1a.

### 3.3. H NMR Analysis and Time-dependent Monitoring of Secondary Aldehydic LOPs in Thermally-stressed CLOs

**Figure 2:**
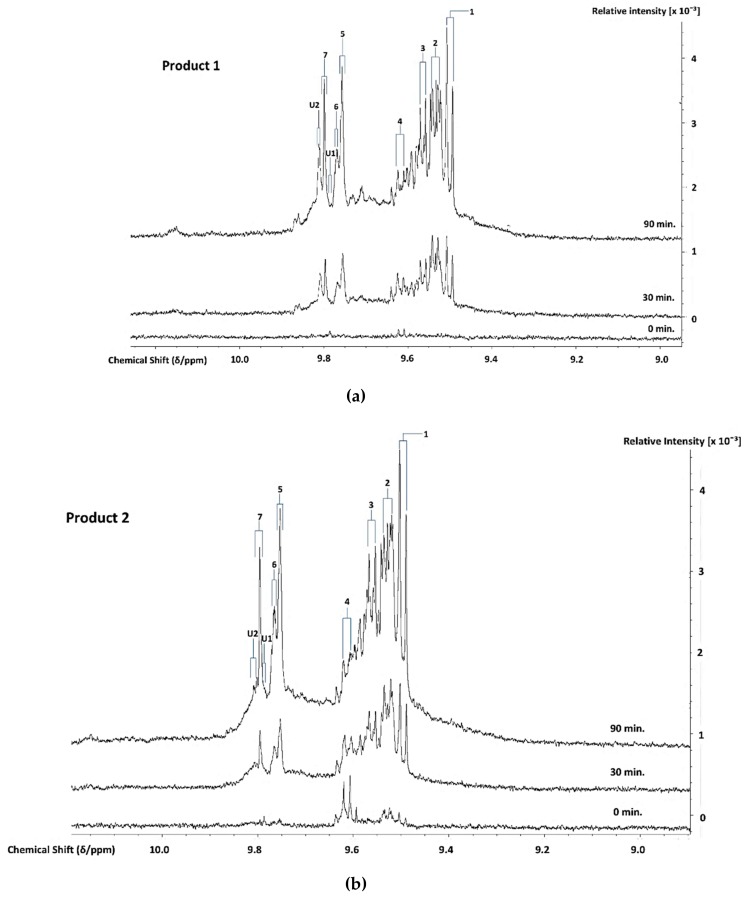

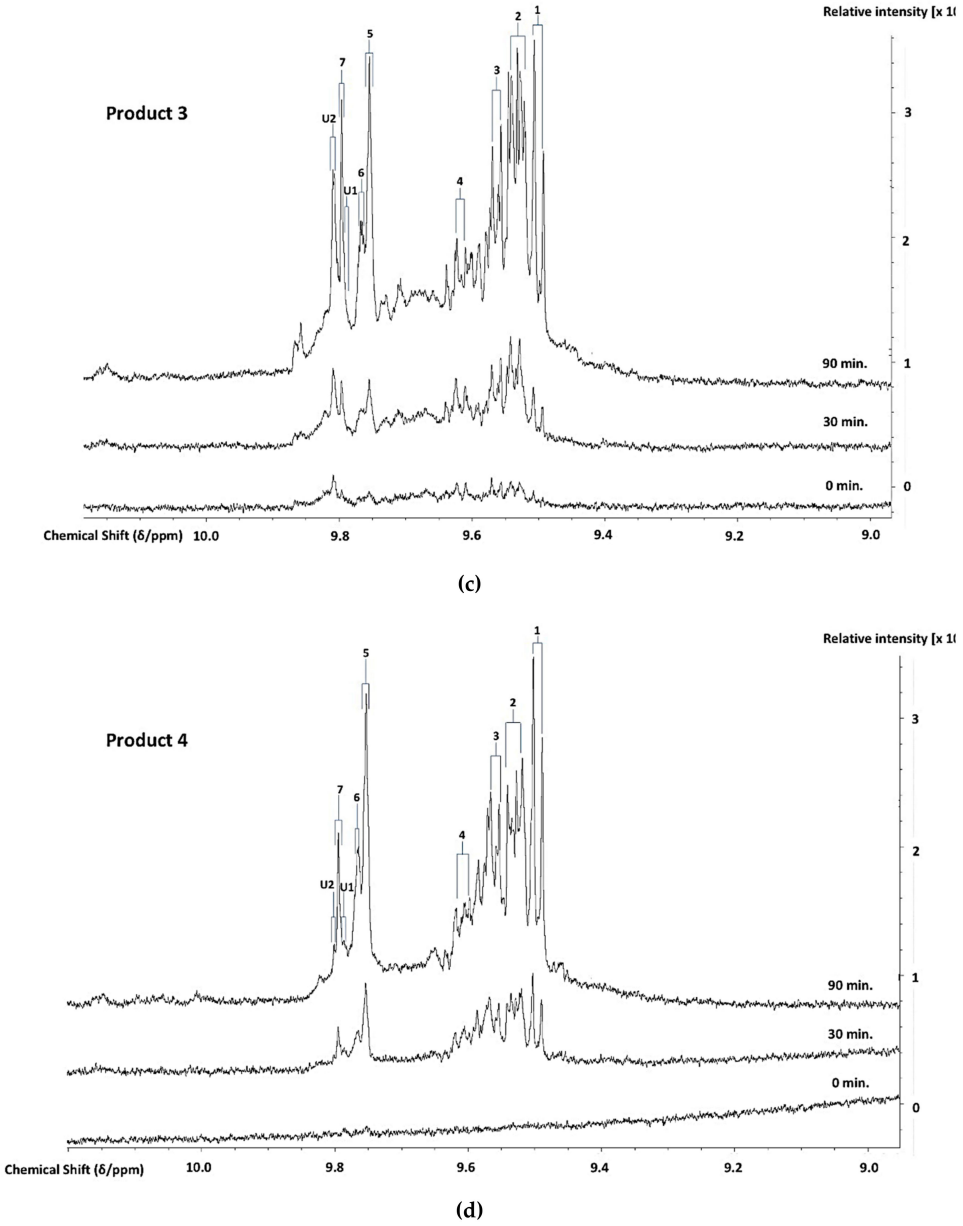
**600 MHz ^1^H NMR Profiles of Aldehydes Generated During TORA TSEs.** Expanded aldehydic-CHO proton (8.95-10.25 ppm) regions of the 600 MHz ^1^H NMR spectra of (**a**) Product 1, (**b**) Product 2, (**c**) Product 3, and (**d**) Product 4 CLOs exposed to TSEs at 180 °C for periods of 0, 30, and 90 min. Typical spectra are shown (the right-hand side ordinate axes indicate the relative intensities of profile resonances). Abbreviations: Number labels correspond to the -CHO function resonances of 1, *trans*-2-alkenals; 2, *trans*,*trans*-alka-2,4-dienals; 3, combined 4,5-epoxy-*trans*-2-alkenals/acrolein; 4, *cis,trans*-alka-2,4-dienals; 5, *n*-alkanals; 6, 4-oxo-*trans*-2-alkenals; 7, low-molecular-mass short-chain *n*-alkanals, particularly propanal and *n*-butanal; U1 and U2, unidentified aldehyde classification signals within the δ = 9.78–9.79 and 9.80–9.82 ppm ISBs.

^1^H NMR analysis demonstrated the thermally-induced production of aldehydic LOPs in all CLOs evaluated, and Figure 2 shows partial ^1^H NMR profiles demonstrating the time-dependent production of aldehyde-CHO function signals assignable to a range of these cytotoxic/genotoxic agents when Products 1–4 were exposed to TSEs for periods of 0, 30, and 90 min. (assignments for these signals were ratified by one- and two-dimensional ^1^H-^1^H COSY and TOCSY spectroscopic analyses of each heated oil [38], in addition to the standard addition ‘spiking’ experiments using calibrated authentic standard solutions of these aldehydes in C^2^HCl_3_). These spectra also contained resonances that were assignable to *cis*,*trans*- *and trans,trans*-CHPDs (conjugated diene olefinic multiplet proton resonances located within the 5.40-6.60 and 5.40-6.30 ppm spectral regions, respectively [38,39,40], along with broad -OOH function signals that were located at δ = 8.40–8.85 ppm, which represent both CHPDs and HPMs. These primary LOPs remained detectable in spectra that were acquired at and after the 90 min. TSE time-point. Moreover, relatively low concentrations of these aldehydes and their CHPD precursors were also detectable in two of the unheated CLO products tested, notably low-molecular-mass *n*-alkanals and *cis,trans*-alka-2,4-dienals that are present in the ^1^H NMR profiles of Products 2 and 3.

However, it should also be noted that, at the later time-points (≥ 60 min.), one or more additional doublet resonances evolved within the *trans,trans*-alka-2,4-dienal aldehyde classification ISB (δ = 9.51–9.55 ppm). This signal heterogeneity was especially the case for Products 2 and 3, but less so for Product 4 (Figure 2). In principle, these further signals could arise from differing molecular mass homologues of this class of aldehyde, e.g. a chemical shift distinction between *trans,trans*-hepta-2,4-dienal (molecular mass 110.15 Da) that arises from the peroxidation of O-3 FA sources [41] (of total content 21–27 molar %, Table S1). Specifically, this aldehyde is derived from the fragmentation of the C14- and C16-hydroperoxides of EPA and DHA acylglycerols, respectively. Moreover, *trans,trans*-deca-2,4-dienal (molecular mass 152.53 Da) arises from the peroxidation of linoleoylglycerols (*ca.* ≤ 2–3% (w/w) only). The partial resolution of two sub-classes of these ^1^H signals is conceivable at an operating frequency of 600 MHz.

**Figure 3:**
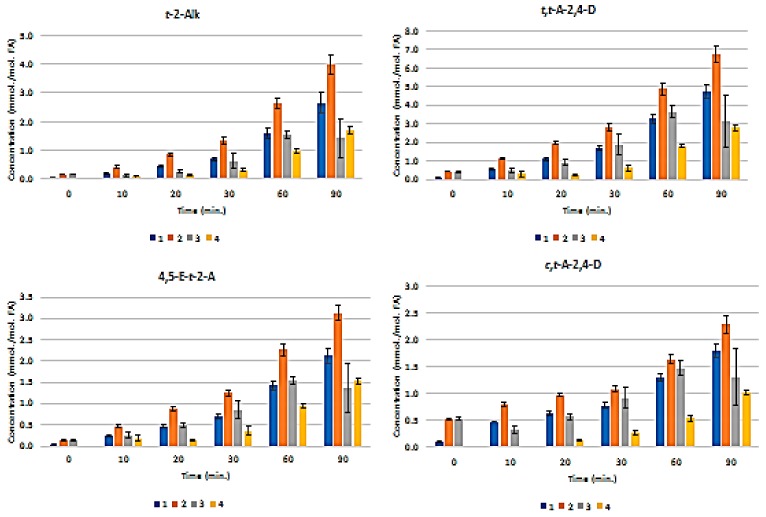

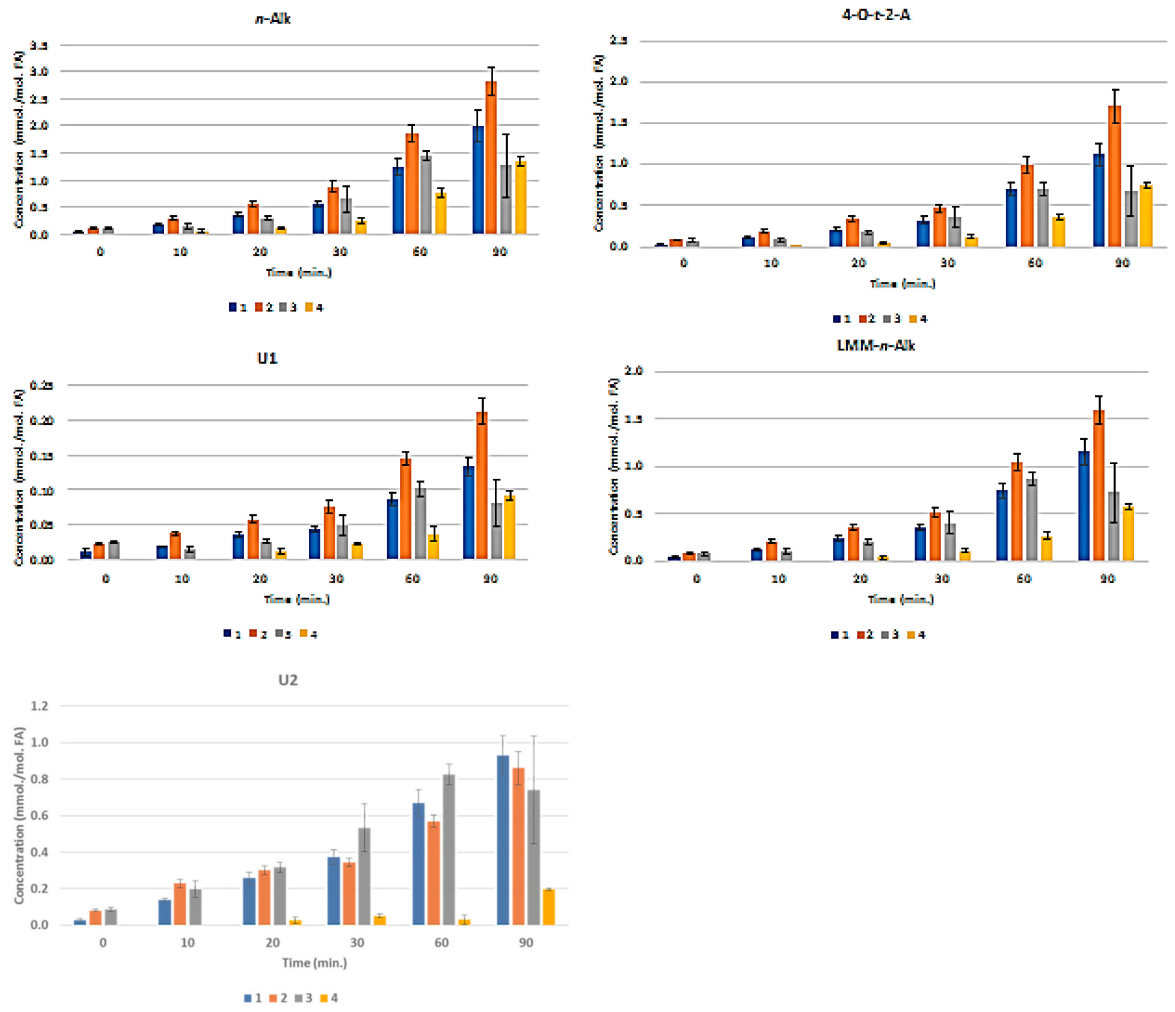
**Mean±SEM Aldehyde Classification Concentrations in CLO Products as a Function of TSE Duration.** Time-dependence of mean ± SEM concentrations of secondary aldehydic LOPs (mmol./mol. FA) generated in CLO products when exposed to TSEs at 180 °C for periods of 0–90 min. Aldehydes monitored were *trans*-2-alkenals (abbreviated *t*-2-Alk); *trans*,*trans*-alka-2,4-dienals (*t,t*-A-2,4-D); combined 4,5-epoxy-*trans*-2-alkenals and acrolein (4,5-E-/Acr); *cis,trans*-alka-2,4-dienals (*c,t*-A-2,4-D); *n*-alkanals (*n*-Alk); 4-oxo-*trans*-2-alkenals (4-O-*t*-2-A); low-molecular-mass *n*-alkanals (*n*-Alk); unidentified aldehyde classifications (U1 and U2). Colour codes 1, 2, 3, and 4 in the abscissa axis panels correspond to designations of the CLO products.

Figure 3 presents the plots of mean ± SEM total FA-normalised concentrations of aldehydes for all nine classes of them. These results show that the pre-fermented Product 4 generates significantly lower levels of these secondary LOPs than those found in Products 1–3 when exposed to TSEs. For example, at the 30 min. TSE time-point, the highest levels of *trans*-2-alkenals, *trans,trans*-alka-2,4-dienals, and *n*-alkanals formed in Products 1–3 were *ca.* 1.3, 2.8, and 0.9 mmol./mol. FA, respectively, but only *ca.* 0.3, 0.6, and 0.25 mmol./mol. lipid, respectively, were found in Product 4. Only a very limited amount of these LOPs were formed in Product 4 at the 10 and 20 min. sampling time points.

Univariate ANCOVA of the experimental data acquired (Equation 1) revealed very highly significant differences between (1) the CLO products investigated (*p <* 10^-9^), and (2) heating time-points (*p* < 10^-9^) for all of the aldehydic LOP-CHO function ISBs monitored, except for the ‘between-time-point’ effect for *n*-alkanals, which had a *p* value of 9.12 x 10^-9^. Moreover, the CLO product x sampling time-point interaction effect was also very highly significant for all determined aldehyde ISBs (*p* < 10^-9^ for *trans*-2-alkenals; 2.63 × 10^-7^ for *trans,trans*-alka-2,4-dienals; 1.78 × 10^-8^ for 4,5-epoxy-*trans*-2-alkenals; 3.66 × 10^-3^ for *cis,trans*-alka-2,4-dienals; 1.95 × 10^-6^ for *n*- alkanals; 2.72 × 10^-8^ for 4-oxo-*n*-alkanals; 7.18 × 10^-8^ for low-molecular-mass *n*-alkanals; and 2.44 × 10^-8^ and 1.16 × 10^-6^ for the unidentified aldehydes U1 and U2, respectively). The mean square estimates for the ‘between-replicates’ components of variance were found not to be statistically significant for any of the ISBs evaluated (*p* = 0.067–0.17), as expected in view of the high level of reproducibility of the acquired results.

Figure S2(a) displays a heatmap diagram showing the time-dependent generation of all nine classes of aldehydes in all four CLO products exposed to the above TSEs (mean levels of *n* = 3 replicates are plotted for each time-point). Agglomerative hierarchal clustering (AHC) provided valuable molecular information regarding the primary sources of aldehydic LOPs and the mechanism of their generation. For example, *trans*-2-alkenals and *n*-alkanals derived from both CHPD and hydroperoxymonoene (HPM) precursors, and 4,5-epoxy-*trans*-2-alkenals from the oxidation of relatively prevalent *trans, trans*-alka-2,4-dienals [26,27].

The U1 and U2 high-frequency ISB region -CHO function multiplet resonances may arise from further aldehydes/aldehyde classifications that are not generally encountered in the ^1^H NMR profiles of thermally-stressed vegetable-derived culinary oils [26], e.g. those derived from the peroxidation of O-3 FAs. These include 2,4,7-decatrienal and 3,6-nonadienal from their 9-hydroperoxides, and 3-hexenal from their 12- and 13-hydroperoxides [40]. However, a further unsaturated aldehyde, which is also generated from O-3 FA sources, is *cis, trans*-nona-2,6-dienal (molecular mass 138.21 Da) [40]. An additional possibility is cytotoxic 4,5-dihydroxy-2,3-decenal [41]. The intensity of the U2 signal was approximately six-fold greater than that of U1 in all thermally-stressed CLO samples. Notwithstanding, for Products 2 and 4, this U2 resonance was only marginally detectable at all heating time-points (Figure 3).

Moreover, malondialdehyde (MDA), which is also selectively derived from the thermally-induced oxidative degeneration of O-3 FAs, has been reported to have a -CHO function resonance located at *δ* = 9.72 ppm [42]. However, this assignment was only tentative. In C^2^HCl_3_ solution, the intramolecularly H-bonded *cis*-enolic form of this dialdehyde appears to be the predominant species present [43]. Notably, formic acid, which is a known degradation product of this aldehyde [44], was detectable in three of the Products tested (Figure 1). Similarly, if present in thermally-oxidised CLO products, acetaldehyde, which is another MDA degradation product, will feature in and, hence, contribute towards the total intensity of the low-molecular-mass (LMM) aldehyde ISB (*δ* = 9.79–9.80 ppm).

The major determinant of the very highly significant CLO product × time-point ANCOVA interaction effect was the differing responses in secondary aldehydic LOP generation occurring at each time-point for all secondary aldehydic LOPs monitored. For example, Product 4 exhibited a significantly longer lag period for aldehyde generation than all of the other products tested, and this observation might indeed arise from higher levels of chain-breaking antioxidants and/or aldehyde-trapping products present therein in view of the fermentation episode employed for its production (Section 2.1). Such antioxidant enrichment of this product will presumably involve the leaching of such PUFA peroxidation-inhibiting agents, for example, collagenous agents, ammonia, aromatic amines, such as phenylethylamine, phenols, flavonones, and tannins from the cod liver tissue matrix into the separating liquid oil medium during this process.

Notwithstanding, it should also be considered that the relative evaporative losses of the differing aldehydic LOPs generated, each containing a range of differing boiling-point (b.pt) homologues, may also be partially responsible for the non-additive responses observed [26]. 

*Post-hoc* ANCOVA analysis of the observed ‘between-CLO products’ differences demonstrated that the magnitudes of aldehyde generation were in the increasing orders of product 4 < product 1 ≈ product 2 ≈ product 3 (*trans*-2-alkenals); 4 < 1 ≈ 3 ≈ 2 (*trans,trans*-alka-2,4-dienals); 4 < 1 ≈ 2 ≈ 3 (4,5-epoxy-*trans*-2-alkenals); 4 < 1 < 3 ≈ 2 (*cis,trans*-alka-2,4-dienals); 4 ≈ 1 < 2 ≈ 3 (*n*-alkanals); 4 < 1 ≈ 2 ≈ 3 (4-oxo-*n*-alkanals); 4 < 1 ≈ 2 ≈ 3 (U1); 4 < 1 ≈ 2 ≈ 3 (low-molecular-mass *n*-alkanals); and, 4 < 1 ≈ 2 ≈ 3 (U2). Table S2 provides the probability (*p*) values of all statistically significant differences found in these *post-hoc* ANCOVA analyses. Figure S2b shows plots of the LSM values of the FA-normalised concentrations of each aldehyde classification monitored for each product tested (i.e. those corrected for highly significant differences between TSE time-points).

Minor resonances that were ascribable to the biogenic amines 2-phenylethylamine and tyramine were found to be completely eliminated from the ^1^H NMR spectral profiles of Product 4 when exposed to TSEs of ≥ 10–20 min., and these data indicate their peroxidative consumption during these heating treatments. Section S17 of the Supplementary Materials provides plausible explanations for and a discussion of this. 

### 3.4. H NMR Analysis and Time-dependent Monitoring of Secondary Aldehydic LOPs in Long-term Stored CLOs

Exposure of each of the four CLO products to 42-day storage episodes in the dark at both refrigerator (4 °C) and ambient (23 ± 1 °C, mean±SD) temperatures only revealed a limited level of aldehyde generation, with Products 2 and 3 giving rise to the highest concentrations (mean post-storage levels < 0.4 mmol./mol. FA for all aldehydes monitored at both TDSE regimens, with the exception of higher levels of *cis,trans*-alka-2,4-dienals). Figure S3 in Supplementary Materials) shows the typical ^1^H NMR profiles acquired at the control and 42-day time-points for each storage process for two of the CLOs investigated. Overall, storage-induced elevations in aldehyde concentrations were found to be of the order of two- and three-fold following dark storage at 4 and 23 °C, respectively (overall range 0–320% of those with minimal baseline 0 hr. time-point aldehyde values, and up to 0.12 mmol./mol. FA for those without any detectable at this time-point). These experimental data will be reported in detail elsewhere.

### 3.5. Chemometric Principal Component Analysis (PCA) of the Aldehydic LOP Concentration Patterns Observed at the Extreme (90 min.) TSE Heating Time-point

**Figure 4:**
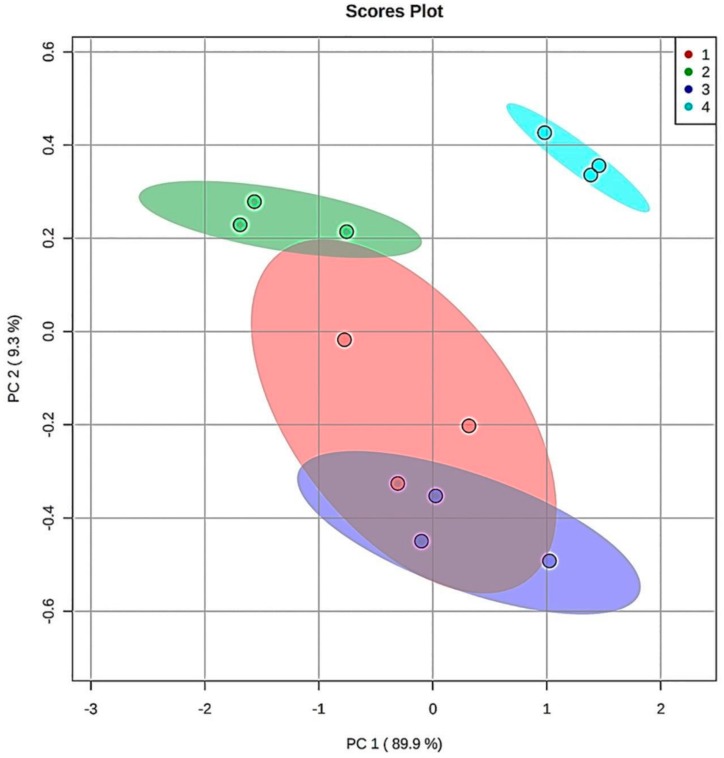
Multivariate Chemometric Clusterings and ROC curve analysis of Aldehyde Classification Patterns Formed in CLO Products at the 90 min. TSE Time-Point. Principal component analysis (PCA) scores plot of PC2 vs. PC1 for a series of nine aldehydic LOP levels (mmol./mol. FA) generated in CLO Products 1-4, and limited to the maximal concentration 90 min. TSE dataset only (*n* = 3 replicates per product). Colour codes for each product are indicated in the top right-hand panel. 95% confidence ellipses (CEs) are displayed for each CLO Product.

Finally, principal component analysis (PCA) was performed on the ^1^H NMR 9–10 ppm aldehydic function dataset region for each of the CLO products exposed to TSEs at 180 °C, but this model only featured samples that were thermally-stressed at the maximal 90 min. sampling time-point. This form of multivariate analysis revealed that, for the nine detectable aldehyde classifications monitored, there were two clear PCs isolable, with the first and second accounting for 90 and 9%, respectively, of the total variance. The first of these (PC1) was loaded with eight strongly-correlated total acylglycerol FA-normalised aldehyde levels (all aldehydes, except that responsible for the U2 signal, loadings scores 0.96–0.99), whilst the second orthogonal (uncorrelated) PC was loaded with only one of these (U2, loading score 0.75). A clear distinction in the patterns of these secondary aldehydic LOP levels between Products 4 and the remaining three CLOs was clearly notable in a corresponding PCA scores plot (Figure 4), and this demonstrated that Product 4 had the highest centroidal score values for both PC1 and PC2. Indeed, the 95% confidence ellipses (CEs) computed demonstrated clear discrimination between Product 4 and all other investigated products.

However, 95% CEs for Products 1 and 2, and 1 and 3, overlapped, although that for Product 1 to only a minor extent. However, there were also clear differences in these aldehydic LOP product patterns between CLO Products 2 and 3, with the former PC having lower and higher centroidal PC1 and PC2 score values, respectively, than the latter. With the exception of the unidentified U2 aldehyde, all of these strongly PC1-loading aldehydes loaded negligibly on PC2 (loading scores of only −0.23 to 0.26). Loading scores values of < −0.40 or > 0.40 were considered to be statistically significant. 

Although the relative orthogonality of the U2 aldehyde classification-loaded PC (PC2) is not simply explicable, this secondary LOP might arise from a differing peroxidation mechanism, or alternatively might be generated from the chemical modification or oxidation of an alternative aldehydic LOP classification during TSE exposure.

A preliminary receiver operating characteristic (ROC) curve analysis was performed in order to evaluate their levels of classification success from the patterns of aldehydic LOP concentrations monitored at the 90 min. TSE time-point in view of the PCA distinction that was achieved between the fermented and unfermented CLO products. The ROC curve arising therefrom (i.e. a plot of true positive *vs.* false positive rates) had area under the receiver-operating characteristic curve (AUROC) values 0.923 rising to 0.974 for models featuring from two to eight aldehyde resonance ISB intensities. However, these values were only statistically significantly greater than the null 0.50 value with eight or nine variables considered in view of the limited sample sizes available (*n* = 3 replicates per product). Therefore, this preliminary analysis provided evidence that this multivariate analysis approach might successfully discriminate between the fermented from unfermented CLO products on the basis of the patterns and concentrations of thermally-inducible aldehydic LOPs generated at the 90 min. TSE time-point.

## 4. Discussion

### 4.1. Antioxidant Potentials of Fermented and Non-fermented CLO Products

Overall, the results acquired in the present study demonstrated that Product 4 offered a very highly statistically-significant level of resistance against thermally-induced oxidative damage to UFAs (particularly EPA and DHA) than those of the non-fermented, albeit unrefined Products 1–3 when evaluated with respect to the concentrations of secondary aldehydic LOPs that were generated as a function of TORA TSE duration. These observations are conceivably explicable by the presence of much higher levels of lipid-soluble chain-breaking antioxidants, such as phenols, phenolic amines, aliphatic amines, flavanones, and/or tannins in this product. Such antioxidants arise from the fermentation process that was employed in its commercial production (Section 2.1). For example, biogenic amines are generated from the microbial degradation of endogenous fish liver proteins, peptides and amino acids, and phenolic agents, which are potentially derived from hydrolysis of their glycoside derivatives. However, further, albeit unexpected lipid radical scavengers, i.e. relatively high concentrations of variable molecular mass collagenous agents (including their hydrolytic degradation products), which also arise from cod liver fermentation, appear to account for the majority of the antioxidant activity that is displayed by this product. Similarly, it is also conceivable that the fermentation product ammonia might provide a significant contribution towards such lipid radical-trapping antioxidant activities. In principle, all of the above agents have the ability to (1) suppress the thermally-stimulated, autocatalytic, self-propagating peroxidation of CLO PUFAs, predominantly that of the very highly-susceptible O-3 FAs DHA and EPA; (2) block the degradation of PUFA-derived CHPDs to aldehyde fragments; and/or, (3) directly react with and, therefore, chemically consume these aldehydic LOPs. However, the capacities of such antioxidants to act in these manners is, of course, critically dependent on their available CLO concentrations, in addition to their abilities to participate in these processes, and the effective rates of each reaction involved. Section 4.4, below, discusses the potential mechanisms for such antioxidant activities. The availability of the above CLO-containing antioxidants, and their protective capacities in Product 4, are supported by the observation of highly elevated, but variable ORAC values therein (ranging from 43 to as high as 162 mmol. trolox equivalents/kg), which were much greater than those that were determined in the other ‘natural’ (unrefined), but unfermented products investigated. Section S16 of the Supplementary Materials provides a review of the potential contributions of Product 4’s low-molecular-mass phenolics, flavanones, biogenic amines, and polyphenolic tannins towards its superior ORAC values, albeit relatively minor ones.

However, the extremely high ORAC values that were found for Product 4 cannot simply be accounted for by the above series of phenolic antioxidants, biogenic amines, flavanones, and tannins, etc. found, since their maximal total (summed) concentration (estimated to be ≤ 8 mmol./kg) is approximately < 12-fold lower than the mean value of this peroxyl radical-trapping capacity. Therefore, this unusually high ORAC index might be significantly or even substantially influenced by this product’s overall total protein (predominantly collagen and collagen hydrolysate peptide) content. Indeed, Ninfali and Aluigi [45] determined this parameter in both whole and deproteinised blood plasma samples that were collected from a range of mammals and avies, the former including humans, and reported that the protein-free specimens only offered 5%–20% of the protection afforded by protein-containing, intact equivalents. Moreover, for most species examined, plasma proteins and lipoproteins accounted for 85%–90% of total peroxyl radical-scavenging capacity. In a further study, the mean ± SD total and deproteinised ORAC values of human blood serum were found to be 3.12 ± 0.15 and only 0.55± 0.034 mmol. trolox equivalents/kg, respectively [46].

Durazzo and Lucarini (2019) recently outlined recent updates on the isolation, properties, and power of antioxidants [47]. This Editorial focuses on new developments in the area of methodological approaches to determining these properties in a range of extractable and non-extractable antioxidants, with special reference to multidisciplinary and innovative experimental designs. Comparisons of conventional extraction technologies with those of newly-developed, more advanced ones, along with analytical techniques available, were considered in depth, especially in relation to green chemistries and technologies. Notably, Nemes et. al. [48] have described a novel method for the extraction of non-extractable membrane-, protein-, and fiber-bound procyanidins. Additionally, a novel HPLC approach for the detection and monitoring of nitric oxide (NO^●^) scavengers that are present in complex plant sources was outlined by Fraisse et. al. [49]; this method was employed for the analysis of *Aloysia triphylla* leaves.

One key emerging focal point in antioxidant research is the coupling of ‘state-of-the-art’ analytical/bioanalytical techniques, such as high-resolution NMR, Fourier-transform infra-red (FTIR), LC-MS, or inductively-coupled plasma-mass spectrometric (ICP-MS) analyses with multivariate chemometrics techniques. An example is that performed in the present study to distinguish between the patterns of aldehydic LOPs produced from the thermally-induced peroxidation of PUFAs present in fermented and non-fermented CLOs (Figure 4). For example, Kock et. al. employed such a strategy [50] to explore the geographical origin of black tea samples; these researchers used secondary metabolite levels and the oxygen radical-scavenging activities of these products as predictor variables, and then applied PCA and univariate ANOVA techniques, etc., to seek, validate, and verify discriminatory signatures for these samples. Moreover, Anonkwuni et. al. [51] explored the spectroscopic and antioxidant properties of both extractable and non-extractable phenolic species that are present in *Terminalia sericea* Burch, and found that the non-extractable forms of these antioxidants contributed less so than the extractable ones.

### 4.2. Detection of Collagen and Its Hydrolytic Degradation Products in Fermented CLO: Roles as Antioxidants

The hypothesis that the high collagen/collagen degradation product levels found in Product 4 predominantly account for its very high ORAC values was fully supported, firstly by the ^1^H NMR detection of a major, highly-intense, added ^2^H_2_O-extinguishable and, hence, deuterium-exchangeable very broad protein/polypeptide-CO-NH- function resonance in ^1^H NMR spectra acquired on it (Figure 1), and which was absent from the corresponding profiles of all other CLOs tested. Secondly, the complete disappearance of this broad protein/polypeptide signal following exposure of this product to peroxyl radical- and aldehyde fragment-generating thermal stressing episodes at 180 °C also corroborates its predominant collagenous/gelatinous identity.

It should also be noted that the amide-CO-NH- function proton of the drug phenacetin in C^2^HCl_3_ solution has a broad ^1^H NMR resonance at *δ* = 8.1 ppm (Δ*v*_½_ ca. 15 Hz) [52]. The much smaller Δ*v*_½_ value of this drug arises from its low-molecular-mass: corresponding values of > 400 Hz are commonly observed for the composite peptide-CO-NH- nuclei resonances of unfolded or partially-unfolded small proteins, and which are comparable to those that are monitored here for Product 4’s collagenous species (≥ 250 Hz) [53]. However, the ^1^H NMR spectrum of formamide in acetonitrile-d_3_ has two broad overlapping -CO-NH_2_ function resonances that span the 7.85–8.40 ppm chemical shift range in view of their coupling to ^14^N nuclei and NMR exchange processes [54], as observed for ammonia and ammonium ion ion-pair complexes in C^2^HCl_3_ solution in this study.

Unfolded proteins with short T_2_ values have very broad -CO-NH- peptide ^1^H resonances (with many contributory ^1^H nuclei for each signal observed), as would be expected to be the case when present in a ‘foreign’ organic solvent medium, such as C^2^HCl_3_. In such cases, the chemical shift dispersion is poor, which leads to very broad composite signals; resonances are sharper and more dispersed for correctly folded small proteins in aqueous media [33]. Section S18 of the Supplementary Materials discusses the extraction of bioactive proteins and peptides into organic solvents from aqueous media, and their solubilities and enzymatic activities therein, whereas Section S19 outlines the novel antioxidant properties of usually discarded collagenous marine products in detail.

Strong positive correlations were found between its intensity and the total protein content of Product 4, consistent with the collagenous polypeptide/peptide source of this broad CLO resonance. Likewise, there was also a strong positive correlation between the ORAC value for this CLO and its protein/polypeptide content. These Product 4 ORAC values are comparable to those that are found for a wide range of antioxidant-rich herbs and spices, for example the total values of this parameter of 320, 140, 140, and 48 mmol. trolox equivalents/kg have been reported for sage, peppermint, oregano, and fresh basil, respectively [55]. Interestingly, Nagatsuka et. al. [56] found that collagen-rich bream fish scale extracts had substantial peroxyl radical-scavenging antioxidant activity, which arose from gelatin, a collagen hydrolysis product, which is present in the tested samples.

Intriguingly, Deyl et. al. [57] found that collagenous material was extractable into a 2:1 chloroform:methanol solvent system from insoluble calf skin collagen. Therefore, it is readily conceivable that selected lipophilic polypeptides, and/or peptide fragments released from cod liver collagen via the fermentation process featured in its manufacture are soluble in and, hence, retained by Product 4’s liquid oil medium. Such chloroform/methanol-extractable collagenous material was found to be rich in glycine, alanine, aspartate, and glutamate [57], and such low-molecular-mass agents are very likely to be more resistant to thermally-induced damage than are folded or unfolded polypeptides. 

It also remains a possibility that stacked or polymeric tannins, or alternative polyphenolic species, may also partially contribute towards this very broad resonance, although this is unlikely in view of the low levels of these agents detectable in this product when expressed relative to that of its tentative estimated single proton-equivalent value, i.e. ca. 0.4 mol./kg.

### 4.3. Detection and Antioxidant Potential of Ammonia in the Fermented CLO Product

Ammonia was also detected in our antioxidant screens of Product 4 at levels of up to 6 mmol./kg. This fermentation product, which is presumably present as charge-neutralised ammonium cations complexed with suitable anions, e.g. free FA carboxylate functions as ion-pair complexes in such lipophilic CLO/C^2^HCl_3_ media, may also have the capacity to offer significant chain-breaking antioxidant protection of PUFAs against peroxidative damage. It has been previously reported that the mole fraction solubility of ammonia (as NH_3_) in chloroform is 0.1–0.2, with ca. 70% of it being in the free form, and the remainder as a 1:1 H-bonded complex with this solvent [58]. Therefore, it is anticipated that NH_3_ will also have significant solubility in edible oils, such as CLOs. Although only a limited amount of information on this is available in the scientific literature, Section S20 of the Supplementary Materials outlines the radical-scavenging antioxidant actions of ammonia and their prevalence in fermented foods.

### 4.4. Multifunctional Mechanisms of Actions of Fermented CLO Antioxidants and Aldehyde-consuming Agents

Equations (2)–(5) outline the radical-scavenging antioxidant actions of ammonia, and their prevalence in fermented foods, the first of which (Equation (2)) indicates the direct consumption of PUFA-derived carbon-centred pentadienyl radical species (or corresponding MUFA-derived trimonoenyl ones) by, for example, the primary amine (-NH_2_) functions of 2-phenylethylamine or tyramine, and/or those that are present in collagenous polypeptides; the second and third (Equations 3 and 4, respectively) involving the direct scavenging of lipid alkoxyl and peroxyl radicals (LO^●^ and LOO^●^, respectively) by phenolic antioxidant-OH functions such as those in tyramine, flavonones and/or tannins, and/or collagenous hydrogen atom donors; and finally, a combination of two antioxidant-derived radical species (Equation (5)). In these equations, RH indicates an antioxidant H^●^ donor species, *e.g.* those of amine-NH_2_ or phenolic-OH functions, collagenous proteins, and polypeptides representing rich sources of these. Moreover, the ample availability of labile peptide bond H^●^ donors in the above collagenous agents also potentially serve as effective antioxidant functions, since amide groups that are present in low-molecular-mass agents can also exert potent anti-radical activities [59].

L^●^ + RH → LH + R^●^ (2)

LO^●^ + RH → LOH + R^●^ (3)

LOO^●^ + RH → LOOH + R^●^ (4)

2R^●^→R_2_ (5)

Additional factors for consideration are (1) the direct consumption (i.e. ‘mopping-up’) of the -CHO functional groups of saturated and unsaturated aldehydic LOPs, with the available amino groups of biogenic amines being detectable in Product 4 via reactions involving the (primary) generation of Schiff base adducts (Equation (6)) [60], and (2) the ability of α,β-unsaturated aldehydes to undergo Michael addition-type reactions with the above amines (Equation (7)) [61], a process that involves nucleophilic attack of these agents at the electrophilic C-3 positions of *trans*-2-alkenals, and *trans,trans-* and *cis,trans-*alka-2,4-dienals, for example.

R_1_-NH_2_ + R_2_-CHO ↔ R_1_-N=CH-R_2_ + H_2_O (6)

R_1_-NH_2_ + R_2_-CH=CH-CHO → R_2_-CH(NH-R_1_)-CH_2_-CHO (7)

Moreover, in principle, collagen, its hydrolytic degradation products, and any further proteins and peptides that are present in Product 4 will also be able to take part in such reaction schemes through the reactivities of selected aliphatic amino acid side-chain-NH_2_ functions available (e.g. those that are present in lysine residues), or to a much lesser extent, their N-terminal-NH_2_ functions. Similarly, any free (i.e. non-NH_4_^+^) ammonia detectable in Product 4 might also conceivably neutralise any toxic aldehydes generated therein (as NH_3_ in place of R_1_-NH_2_ in Equations (6) and (7). For the Schiff base reaction scheme (Equation (6)), both primary and secondary, but not tertiary amines, are reactive towards aldehyde-CHO functions, firstly forming corresponding imines and enamines respectively [60]. Additional details regarding the antioxidant properties of biogenic amines are available in Section S21 of the Supplementary Materials.

A further plausible explanation for the novel oxidative resistance of Product 4 against thermally-mediated peroxidative damage to its UFAs relates to the differences observed between the highly peroxidatively-susceptible O-3 FA contents of the CLO products investigated. However, only small differences were found between the ^1^H NMR-determined total O-3-FAs, EPA, DHA, total PUFA, and total MUFA contents of these products (Table S1). Notwithstanding, it should be noted that the [DHA]:[EPA] concentration ratios of products 1, 2, 3, and 4 were found to be 1.06, 1.03, 1.02, and only 0.60, respectively, and these indices may also be partially responsible for the differences that were observed in thermally-mediated aldehydic LOP generation between Product 4 and the remaining CLOs. Indeed, the relative peroxidative susceptibilities of EPA and DHA are 3:4 [62].

The direct observation of singlet ^1^H NMR resonances ascribable to both the DTBHQ antioxidant (added to suppress artefactual peroxidation episodes occurring during sample preparation and storage periods) and its corresponding benzoquinone oxidation product was an additional important incidental observation that was made during the course of these investigations (Figure 1). Hence, this observation, specifically the relative intensities of these two ^1^H NMR signals, appears to provide a novel means of monitoring the ability of such synthetic phenolic antioxidants to intercept and, therefore, prevent artefactual peroxidation that occurs during these laboratory periods, and further experiments to evaluate this are currently underway.

### 4.5. Advantages Offered by NMR-based TORA Analysis of CLO LOPs

This investigation also confirmed that high-resolution ^1^H NMR analysis has the ability to determine the relative oxidative resistivities of marine oil products when exposed to thermal stressing episodes according to our TORA TSE protocol, or when stored for increasing time periods at ambient or refrigerator temperatures. As noted, this analytical strategy serves as an alternative to the accelerated Rancimat® method [25], and it provides a very high level of detailed molecular information regarding the identities of secondary aldehydic LOP classes, and also their peroxidised FA sources (e.g. acrolein, malondialdehyde, and low-molecular-mass *n*-alkanals, such as propanal derived from the fragmentation of O-3 FA hydroperoxides only). 

Of further importance is the observation of low levels of aldehydes in two of the control (unheated) CLO samples investigated here, i.e. particularly for Products 2 and 3, and these agents may arise from lengthy periods of storage at ambient temperature, elevated product extraction temperatures, and/or excessive exposure to atmospheric O_2_ or light during commercial processing and manufacturing episodes.

### 4.6. Nutritional and Health Significance of CLO LOPs and Chain-breaking Antioxidants

Variable concentrations of toxic LOPs, such as aldehydes detectable in marine oil provisions, may serve to explain the variabilities in research outcomes that were observed in studies focused on their positive health benefits [63,64]. Indeed, such oxidized marine oils may have altered biological activities and/or adverse toxicological properties, which, in turn, may negate any positive health effects that are offered by O-3 FAs and their PL derivatives, i.e. such oxidation might render products containing them ineffective or even harmful to humans. Therefore, the results acquired in this investigation concerning the relative peroxidisabilities of the products tested may have significant implications regarding the performance of clinical trials focused on the screening of such health benefits. Indeed, the authors thoroughly recommend the prior reporting of the results of assays for UFA-derived LOPs, since this will serve to facilitate our understanding of the biological and health effects of such marine oil products, and also the potentially adverse health properties of peroxidised O-3 FAs. 

In view of the powerful lipid radical-trapping antioxidant activities of the fermented CLO product that is investigated here, it is anticipated to have an extended shelf-life over those of products with lower or much lower ORAC values (preliminary results acquired that are shown in Figure S3 indicate this). Hence, further experiments to explore this are required. Although the ability of chain-breaking and/or alternative activity antioxidants to exert their protective functions against CLO UFA peroxidation at the high TORA temperatures employed here (Section 3.3) remains somewhat limited, either because of their volatilities (e.g., selected biogenic amines) and/or their thermal instabilities (e.g., tocopherols and carotenoids), such actions, which are much more readily exerted at ambient or lower storage temperatures, are expected to be prolonged, molecularly extensive, and multifarious. However, such elevated temperatures will, of course, markedly enhance the rates and thermodynamic favourabilities of at least some of the reactions involved, for example, the direct reactions of collagenous peptide degradation products, biogenic amines, and/or ammonia with aldehydes.

It should also be noted that the evaluated fermented CLO product also offers some potential for use in animal nutrition or fish farming purposes and, therefore, the enhanced peroxidative resistivity and, by implication, the extended shelf-life of Product 4 will serve as a major advantage for such applications. Currently, the latter aquacultural use accounts for ca. 50% of fish food requirements; this figure is expected to increase to 60%–70% by 2030 [65]. In view of the inability of marine, carnivorous fish species to desaturate and elongate linoleic and α-linolenic acids to longer chain PUFAs, the farming of such species requires the external provision of DHA, EPA, and arachidonic acid through food sources. Hence, currently, there is escalating demand for these essential FAs. Such farmed fish now represent a highly popular protein source alternative to that available from other animals [66].

The production of chain-breaking antioxidant agents, such as biogenic amines, phenols, flavanones, tannins, and hydrolytic collagenous degradation products during fermentation episodes, is markedly dependent on the microorganisms employed, cultivation media, durations, temperature, and pH values, along with the availability of stimulators, inhibitors, and atmospheric O_2_ [23]. However, in this research area, bioactive agents that arise from fermentation are often newly discovered, and careful design and control of this process could, in principle, be customised so as to enhance the levels and bioaccessibilities of such compounds. Indeed, some generated peptides have valuable positive health effects, and other agents arising therefrom exert powerful microbicidal properties [24]. However, this largely remains an unexplored area of much nutraceutical potential.

From an additional consumer nutritional viewpoint, it should also be noted that the most frequent consumer complaint regarding the intake of marine oils, or their encapsulated supplements, is that a high percentage of recipients experience what is described as a ‘fishy burp’ [67]. Such ‘burps’ may occur rapidly (within 15 min.), or alternatively within several hours following ingestion if the rate of gastric emptying is very slow (for marine oil capsules, this is dependent on the rate of capsular dissolution and consequent oil release in the stomach). Although by no means a serious adverse effect, steps have been taken to overcome this problem, including entericoated capsule intake, freezing the fish oil source prior to use, or ingesting it immediately prior to retiring at bedtime in the evening [67]. In view of these observations, it might be useful to investigate and compare the relative incidences, frequencies, prolongation, and time-dependencies of such ‘fishy burp’ episodes occurring in cohorts of participants receiving fermented, non-fermented natural (unrefined), and/or refined CLO products.

## 5. Conclusions

In conclusion, this study demonstrated that statistically significantly lower levels of cytotoxic and genotoxic aldehydes were formed in the fermented Product 4 than those that were determined in Products 1–3 when exposed to TORA TSEs. ^1^H NMR and additional analytical techniques confirmed that Product 4 contained significant levels of peroxidation-blocking, chain-terminating antioxidant fermentation products, including flavanone, phenolic and tannin antioxidant species, much higher ^1^H nucleus-equivalent concentrations of collagen and its hydrolytic degradation products, and ammonia, together with significant levels of primary and secondary biogenic amine functions (ca. 1.4 mmol./kg total low-molecular-mass, non-collagenous amine groups). Those containing available amine and peptide functions have the ability to directly consume aldehydic LOP toxins. A very broad ^2^H_2_O-extinguishable and heat-sensitive resonance that was present in the ^1^H NMR profiles of Product 4, but not those of Products 1-3, provided further strong evidence for the availability of collagenous and, to a lesser extent, ammoniacal agents in Product 4, which might also act as powerful peroxyl, alkoxyl, and carbon-centered radical scavenger(s). However, the concentrations of α-TOH and carotenoids available in Product 4 were considered to be insufficient to effectively compete for LO^●^ and LOO ^●^ peroxidation radical adducts in thermally-stressed samples of it, i.e. mean levels of only 130 µmol./kg and 1.86 mg/kg, respectively; the former concentration was similar to those found in the other (unfermented) products tested. The determination of ORAC values confirmed that Product 4 had a markedly higher peroxyl radical-scavenging capacity than those of Products 1–3. Therefore, the natural fortification of such products with a series of such chain-breaking antioxidants and/or collagenous agents via fermentation processes appears to powerfully impair the deleterious peroxidation of PUFAs in such products, and, hence, enhance their peroxidative stability. A preliminary report of this work is available in [68].

Moreover, this investigation also revealed that high-resolution ^1^H NMR analysis served as an extremely valuable technique for evaluating the peroxidative resistivities of a series of natural CLO products when exposed to high-temperature TSEs, or alternatively long-term storage episodes at 4 and 23 °C, and the analyte concentration datasets provided by a wealth of alternative analytical methods supported the acquired results. This multicomponent analytical TORA approach offers many advantages over the established accelerated Rancimat® method [25] in terms of its ability to provide information on the molecular nature and the levels of a wide range of LOP classes (e.g., ≥ 11 classes of aldehydic LOPs derived from lipid hydroperoxide fragmentation), and hence facilitates the deduction of valuable CLO-specific mechanistic information regarding the peroxidisabilities of UFAs therein, particularly O-3 FAs. This analytical strategy was also found to provide considerable valuable information regarding the concentrations and status of antioxidants present in the CLO products evaluated, i.e. it had the ability to monitor the biogenic amines 2-phenylethylamine and tyramine in the fermented CLO product, and confirmed their peroxidative consumption when exposed to TORA TSEs. The developed method is also readily applicable to investigations of the peroxidative resistivity of a wide range of further edible oil food products, particularly vegetable-derived frying oils, such as sunflower or corn oils. 

Unlike sample-destructive and often time-consuming analytical approaches, such as GC or gas chromatographic-mass spectrometry (GC/MS), high-resolution NMR spectroscopy offers major analytical advantages over alternative approaches, most especially since it permits the rapid, simultaneous, virtually non-invasive, and non-destructive evaluation of a multitude of both major and minor agents that are present in complex, multicomponent CLO samples, and culinary oils in general. Indeed, we applied this technique in the current study to successfully determine the acylglycerol FA composition/status of marine oil products, and the nature and concentrations of a range of minor antioxidants and nutrients, such as CDPs, biogenic amines, etc. However, FTIR analysis, which is now widely considered as a ‘green’ analytical technique [69], is much more convenient to use than high-resolution NMR analysis, and it also offers many applications to the assessment of CLO product authenticities [70], and the peroxidation status of edible oils in general [71]. Notwithstanding recent developments in the operation of near-portable low-field benchtop NMR spectrometers for such culinary oil analysis purposes also provides considerable analytical advantages [72], including an improved molecular specificity than that of FTIR approaches.

A limitation of the current study is that the boiling-points (b.pts) of some key aldehydes are below the TSE temperature that was employed for these investigations. For example, propanal and acrolein arising from ω-3 FA peroxidation (the latter also from oxidation of triacylglycerol glycerol backbones) have b.pts of 49 and 53 °C, respectively. Therefore, the aldehyde classifications determined in this investigation only represent the residual fractions of those present in CLO products at the specified TSE sampling time-points. An additional limitation is that the activities of the selected antioxidants detectable in this study may be limited somewhat because of their thermal instabilities at the high TSE temperature employed, for example, those of carotenoids, tocopherols, and possibly CDPs, and, in some cases, also their volatilities, e.g. selected biogenic amines and tocopherols, with the latter having b.pt values close to 180 °C (200–220 °C).

## Figures and Tables

**Table 1:** Nutrient and Antioxidant Concentrations of cod liver oil (CLO) Products 1–4.

Nutrient/Antioxidant	Concentration Units	Product 1 (*n* = 4)	Product 2 (*n* = 4)	Product 3 (*n* = 4)	Product 4 (*n* = 5)
Total Flavonoids*	mg/kg	nd	nd	nd	nd
Total Flavanones*	mg/kg	nd	nd	nd	896 ± 444
Total Phenolics*	mg/kg	nd	nd	nd	79.7 ± 9.5
Tannins*	mg/kg	nd	nd	nd	112.2 ± 150.5
Anthocyanins*	mg/kg	nd	nd	nd	nd
α-Tocopherol*^†^	mmol./kg	0.09 ± 0.03	0.12 ± 0.02	0.12 ± 0.01	0.13 ± 0.09
γ-Tocopherol*	mg/kg	na	na	na	trace
β-Carotene*	µmol./kg	na	na	na	1.49 ± 1.75
Total Carotenoids*^†^	mg/kg	0.79 ± 0.07	0.73 ± 0.02	0.77 ± 0.02	1.86 ± 2.04
Total Chlorophyll	mg/kg	na	na	na	2.04 ± 1.53
Chlorophyll A^†^	µmol./kg	0.23 ± 0.01	0.23 ± 0.02	0.20 ± 0.02	0.23 ± 0.03
Chlorophyll B	µmol./kg	nd	nd	nd	0.42 ± 0.03
Retinol^†^	mmol./kg	0.10 ± 0.01	0.09 ± 0.03	0.16 ± 0.02	0.10 ± 0.06

Table 1. Mean ± standard deviation (SD) concentrations of flavonoids, flavanones, total phenolics, tannins, anthocyanins, tocopherols, carotenoids, chlorophylls and retinol (vitamin A) for 4 or 5 different batches of Products 1–4. * Indicates phenolic antioxidant species. ^†^ Statistical significance of differences between mean values were α-tocopherol not significant (ns); total carotenoids *p* < 0.01 (Product 4 > Products 1 ≈ 2 ≈ 3); chlorophyll A ns; retinol *p* < 0.03 (Product 3 > Product 2 only). Abbreviations: nd, none detectable, i.e. below the quantification limit; na, not assayed. Statistical significance was determined by ANOVA followed by Bonferroni *post-hoc* tests.

**Table 2:** Biogenic Amine Concentrations of Fermented CLO Product 4.

	Batch Number					
Biogenic Amine	1	2	3	4	5	Mean ± SD (Product 4)
2-Phenylethylamine (mmol./kg)	0.71	0.85	0.41	0.14	0.63	0.55 ± 0.28
Tyramine* (mmol./kg)	0.51	0.64	0.31	0.06	0.23	0.36 ± 0.23
Tryptamine (mmol./kg)	0.22	0.15	0.16	0.02	0.05	0.12 ± 0.08
Cadaverine (mmol./kg)	0.13	0.06	0.14	nd	0.04	0.07 ± 0.06
Putrescine (mmol./kg)	0.08	0.06	0.08	nd	nd	0.04 ± 0.04
Spermidine (mmol./kg)	nd	0.03	nd	nd	nd	0.01 ± 0.01
Total Biogenic Amine Functions** (mmol./kg)	2.1	2.1	1.5	0.2	1.0	1.39 ± 0.78

Table 2. Biogenic amine concentrations, together with that of total biogenic amine function (mmol./kg), for 5 separate batches of Product 4 (1–5). All these amines were undetectable in all separate batches of Products 1–3 analysed. * Indicates phenolic antioxidant. ** Total biogenic amine function concentrations were determined via a consideration of the number of primary and secondary amine functions in each molecule considered (primary amine functions were the major contributors). All tertiary amine functions were excluded. Mean ± SD concentration values are also provided. Spermine and histamine were undetectable in all four products analysed. Abbreviations: as Table 1.

## Supplementary Materials

The following are available online at https://www.mdpi.com/2072-6643/12/3/753/s1, Figure S1: **(a)** Partial (4.90–9.10 ppm region of) the ^1^H NMR spectra of ^2^H_2_O extracts of Products 1–4. **(b)** Expansion of this spectral region for Product 4. Figure S2: **(a)** Heatmap diagram showing the time-dependent generation of *trans*-2-alkenals (t-2-Alk); *trans*,*trans*-alka-2,4-dienals (t,t-A-2,4-D); combined 4,5-epoxy-*trans*-2-alkenals and acrolein (4,5-E-t-2-A/Acr); *cis,trans*-alka-2,4-dienals (c,t-A-2,4-D); *n*-alkanals (n-Alk); 4-oxo-*trans*-2-alkenals (4-O-E-t-2-A); low-molecular-mass *n*-alkanals (LMM n-Alk); Unidentified aldehyde classifications with resonances located within the *δ* = 9.78–9.79 and 9.80–9.82 ppm ISBs (U1 and U2 respectively) in CLO samples exposed to TSEs. Colour codes for Products 1–4 are shown in the top right-hand side panel. The abscissa axis depicts the CLO brands tested and the TSE heating time at 180 °C (min.), for example 1_00 and 1_90 indicate Product 1 thermally-stressed for 0 and 90 min. respectively. Generalised log- (glog-) transformed aldehyde concentrations (mmol./mol. FA) are shown in the right-hand side y-axis: deep blue and red colourations represent extremes of low and high concentrations respectively. **(b)** Bar diagram plots of least squares mean (LSM) values of each aldehydic LOP classification for each of the 4 CLO products evaluated (colour codes for Products 1–4 are indicated on the abscissa axis; abbreviations for aldehyde classes correspond to those given in **(a)** above). A, B and C represent statistically significantly distinct products or groups of products, whereas AB and BC represent product LSM values which are intermediate between the two products/product groups indicated. Figure S3: Aldehydic (9.0–9.8 ppm) proton regions of the 600 MHz ^1^H NMR spectral profiles of Products 2 and 4 on day 0 (bottom), and after 42 days storage in the dark at ambient temperature (mean ± SD 23 ± 1 °C) (middle) and in a darkened refrigerator at 4 °C (top). Typical spectra are shown. Table S1: 1H NMR-determined mean ± SEM molar % fatty acid contents of products 1–4, specifically total saturated fatty acids (SFAs), UFAs and O-3 FAs, together with those of DHA and EPA. Table S2: Statistical significance (*p* values) of *post-hoc* ANCOVA analysis of the ‘between-CLO products’ source of variation for the experimental model employed (Equation 2).
