# Supplementary material for: Evaluations of the Peroxidative Susceptibilities of Cod Liver Oils by a 1H NMR Analysis Strategy: Peroxidative Resistivity of a Natural Collagenous and Biogenic Amine-Rich Fermented Product
Source: Nutrients. 2020 Mar 12;12(3):753. doi: 10.3390/nu12030753 (PMC7146420; doi:10.3390/nu12030753)
Supplement: Supplementary file 1 [file nutrients-12-00753-s001.pdf]

Article

Evaluations of the Peroxidative Susceptibilities of Cod Liver Oils by a ^{1}H NMR Analysis Strategy: Peroxidative Resistivity of a Natural Collagenous and Biogenic Amine-Rich Fermented Product

Benita C. Percival [1], Angela Wann [1,2], Richard Zbasnik [3], Vicki Schlegel [3], Mark Edgar [4], Jie Zhang [5], Gilbert Ampem [2], Philippe Wilson [1], Adam Le Gresley [2], Declan Naughton [2], and Martin Grootveld [1,*]

[1] Leicester School of Pharmacy, De Montfort University, The Gateway, Leicester LE1 9BH, United Kingdom; P11279990@myemail.dmu.ac.uk, P10388223@365.dmu.ac.uk, philippe.wilson@dmu.ac.uk
[2] Department of Applied and Human Sciences, Kingston University London, Penrhyn Road, Kingston-upon-Thames KT1 2EE, United Kingdom; k1741966@kingston.ac.uk, A.Legresley@kingston.ac.uk, D.Naughton@kingston.ac.uk
[3] Natural Product Analysis Laboratory, Department of Food Science and Technology, University of Nebraska-Lincoln, 1901 N 21st Street, Lincoln, NE 68588-6205, USA; zbasnik@unl.edu, vschlegel3@unl.edu
[4] Department of Chemistry, University of Loughborough, Epinal Way, Loughborough, LE11 3TU, United Kingdom; M.Edgar@lboro.ac.uk
[5] Green Pasture Products, 416 E. Fremont Street, O'Neill, NE 68763, USA; gpplab@greenpasture.org
[*] Correspondence: mgrootveld@dmu.ac.uk; Tel.: +44-(0)116-250-6443

Received: 1 February 2020; Accepted: 6 March 2020; Published: 12 March 2020

Supplementary Materials Sections S1-S15: Non-NMR Analyses of CLO Antioxidants, Phenolics, Flavonoids, Flavonones, Tannins, Biogenic Amines, Anthocyanins, Carotenoids, Chlorophylls, Retinol and Tocopherols, Total Protein, Collagen and its Hydrolytic Degradation Products, Ammonia, ORAC Values, Moisture and Fatty Acid Contents of CLO Products.

S1. Fatty Acid Contents and Peroxidative Susceptibility Indices of Product 4 Batches Determined by Gas Chromatography (GC)

For Product 4, values of 19.4-20.8% (w/w) for total saturated fatty acids (SFAs) (including palmitic acid 11.6-12.4%, and stearic acid 3.0–3.4% (w/w)); 43.5–48.7% (w/w) for total MUFAs (including palmitoleic 10.7–13.1%, and oleic acid 27.2–30.3% (w/w); 30.2–33.7% (w/w) for total PUFAs; 27.6–30.7% (w/w) for total O-3 FAs (including 14.1–15.1% EPA, and 10.4–13.8% (w/w) DHA); 2.43-2.85% (w/w) O-6 FAs (including 0.92–1.06% (w/w) linoleic acid); 27.7–30.3% (w/w) O-9 FAs (including the oleic acid value provided above); and, 1.73–1.99% (w/w) total *trans*-FAs, were found.

From the FA compositions of these oils, their intrinsic peroxidative susceptibility indices (PSIs) were computed, as previously described [58], *i.e.* Equation S1.

PSI = [0.025(% monoenoic FA)] + [1.00(% dienoic FA)] + [2.00(% trienoic FA)] + [4.00(% tetraenoic FA)] + [6.00(% pentaenoic FA)] + [8.00(% hexaenoic FA)] (Eq. S1)

For two typical batches of Product 4, these PSI values were 191.8 and 200.7 from our GC analysis, whereas that computed from corresponding FA levels of a hypothetical typical non-fermented CLO product consisting of 21.0% SFAs, 48% MUFAs, 2% linoleic acid (18:2), 2% linolenic acid (18:3 (n-3)), 1% eicosatetraenoic acid (20:4), 1% octadecatetraenoic acid (18:4), and 12% EPA and 13% (w/w) DHA (with all FA contents within the guideline ranges [S1]), yielded a similar value of 195.2.

S1.1 Method for gas chromatographic (GC) determinations of CLO fatty acid (FA) contents

The CLO FA contents were determined by the AOAC Method 996.06, which involves hydrolytic extraction, methylation, and capillary GC-flame ionization detector (FID) analysis of fatty acid methyl esters (FAMEs) generated therein [S2].

A 100 to 200 mg mass of sample was accurately weighed and dissolved in a 2.00 ml volume of chloroform containing 5.00 mg/mL triundecanoin as an internal standard. To each sample, 100 mg of pyrogallol and 2.00 mL of ethanol were added and the mixtures rotamixed, before finally adding a 10.0 mL volume of 8.3 M hydrochloric acid and then incubating at a temperature of 75ºC for 40 min. whilst shaking at 10 min. intervals. The lipids were extracted with 25 ml of diethyl ether and an equivalent volume of hexane. The solvent was removed under N_2 and then re-collected and transferred to a 5 ml Reacti-Vial containing a mixture of 2.0 ml of diethyl ether and 2.0 ml of chloroform. The solvent was removed under N_2, and then 1.0 ml of toluene and 1.0 ml of 14% (w/v) boron triflouride in methanol were added before capping tight and equilibrating for 40 min. at 90ºC. The samples were allowed to cool to ambient temperature before being combined with a 4.0 ml volume of doubly-distilled water, along with 1.0 g of anhydrous sodium sulphate and 1.0 ml of hexane. The samples were spun to separate the phases and the hexane phase was collected for injection purposes.

FAs were separated and identified with an Agilent 7820A GC system that was equipped with a flame ionisation detector and an Agilent G4567A autosampler. A 1.0 μl portion of the prepared sample was injected onto a Suppelco RT-2560 column. The injection port was maintained at 225ºC and the detector was retained at 250ºC, with the oven starting at 100ºC for 4 min. before increasing to 240ºC at a rate of 3ºC per min., and then holding at this temperature for a 20 min. period. The FA retention times were determined by standard injection, and total FA levels were calculated based on their peak areas expressed relative to that of the internal standard.

S2 ^{1}H NMR Analysis and Sample Preparations

S2.1 Exposure of CLO samples to TORA thermal-stressing and temperature-dependent storage episodes (TSEs and TDSEs respectively), and their preparation for ^{1}H NMR analysis

These experiments were performed by a 'blinded' laboratory researcher. Each 90 min. heating cycle was completed n = 3 replicated sessions for all CLO products investigated. These TSEs involved the heating of a 6.00 ml total volume of CLO in an air-dried 250 ml glass beaker within a silicon oil bath fitted with a thermostat, and maintained at a temperature of 180°C throughout the total duration of heating. Aliquots (0.20 ml) of oil samples were collected at the 0, 10, 20, 30, 60, and 90 min. heating time-points for ^{1}H NMR analysis. Immediately following collection, the lipid-soluble chain-terminating antioxidant 2,5-di-tert-butylhydroquinone (DTBHQ) was added to each CLO sample (0.10 ml of a 50.0 mmol./L solution) in order to suppress the artefactual generation of LOPs during periods of storage, and sample preparation at ambient temperature. The samples were prepared for ^{1}H NMR analysis within 2 hr. of collection, and were then again stored in tight-sealed containers within a light-excluded zone in a freezer at -80°C whilst awaiting analysis.

S2.2 Preparation of $^{2}H_2O$ and $C^{2}H_3O^{2}H$ extracts of CLO Products 1-4 for ^{1}H NMR analysis

The aqueous ($^{2}H_2O$) extracts of all CLO products (n = 3 replicates in each case) were performed by the addition of a 1.00 mL volume of $^{2}H_2O$ to an equivalent volume of oil, followed by thorough vortex mixing and centrifugation (3,500 rpm) at 20°C. A 0.50 mL volume of the lower $^{2}H_2O$ layer was then removed and treated with a 50 μL volume of a solution of 0.05% (w/v) sodium 3-trimethylsilyl-(2,2,3,3-$^{2}H_4$)-1-propionate (TSP), also in $^{2}H_2O$ (2.90 mmol. TSP/L), which served as an internal chemical shift reference and quantitative NMR standard. The $C^{2}H_3O^{2}H$ extracts were similarly obtained, with 1.00 mL added volumes of this NMR solvent being used in place of added $^{2}H_2O$.

S2.3 ^{1}H NMR analysis

The ^{1}H NMR measurements on the above samples were conducted on a 600 MHz Bruker Avance AV-600 spectrometer (Kingston University London facility) operating at a frequency of 600.13 MHz and a probe temperature of 293 K. Typically, the above 2:1 (v/v) CLO:DTBHQ solution admixtures (0.30 ml) were treated with 0.40 ml of deuterated chloroform ($C^{2}HCl_3$) containing 0.34 mol./L tetramethylsilane (TMS), and 0.060 ml of 66.00 mmol./L 1,3,5-trichlorobenzene (1,3,5-TCB): the $C^{2}HCl_3$ diluent provided a field frequency lock, the TMS acted as an internal chemical shift reference (δ = 0.00 ppm), and 1,3,5-TCB (s, δ = 7.20 ppm) served as a further internal reference standard. The residual $CHCl_3$ in the $C^{2}HCl_3$ solvent employed had a chemical shift value of 7.27 ppm.

These solutions were then placed in 5-mm diameter NMR tubes. The typical pulsing conditions were: zg30 pulse program, with two dummy scans; 128 free induction decays (FIDs), each involving 65,536 data points; an acquisition time of 2.66 s; and, a sweep width of 20.55 ppm. Resonances that were present in each spectrum were routinely assigned by a consideration of chemical shift values, coupling patterns, and coupling constants. One- and two-dimensional COSY and TOCSY spectra were acquired to confirm ^{1}H NMR assignments, as previously described [10,11,14].

Carr-Purcell-Meiboom-Gill (CPMG) spectra [24,25] of Product 4 were obtained on a JEOL-ECZ-500 500 MHz spectrometer (University of Loughborough facility) while employing Delta version 5.2.1 software. A total of 64 scans were acquired for these experiments, covering a spectral width of 16.0 ppm with a precisely set 1H P90 of 7.6 μs at 18 dB attenuation. The τ delay was set to 0.9924 ms, and 500 loops were cycled.

Evidence for the source of a very broad amide-CO-N<u>H</u>- function resonance detectable in ^{1}H NMR spectra of one of the products investigated (Product 4) was obtained by the treatment of samples of it prepared for ^{1}H NMR analysis, as outlined in Section S2.1 above (of total volume 0.70 mL, comprising 0.20 mL CLO, 0.40 mL of $C^{2}HCl_3$, and 0.10 mL of DTBHQ antioxidant solution in $C^{2}HCl_3$) with a 10 μL volume of $^{2}H_2O$, and then thorough rotamixing of the admixture prior to transferring to 5-mm diameter NMR tubes for ^{1}H NMR analysis. An alternative approach involved the exclusion of the above TCB internal standard solution to avoid interferences from this internal standard's satellite resonances with the observation of those arising from Product 4's aromatic antioxidants (final volume only 0.60 mL in this case).

The ^{1}H NMR spectra of the 10 μL volume $^{2}H_2O$-treated samples were acquired on a JEOL-ECZR600 NMR spectrometer (De Montfort University, Leicester, UK facility) operating at a frequency of 600.17 MHz. The acquisitional parameters were: acquisition time 1.45 sec; 16 scans; receiver gain of 26; 45° pulse angle; 3.315 μs pulse; repetition time 6.45 s; 16,384 datapoints acquired; and, spectral width 9,000 Hz.

Micro-extractions of the above 0.70 mL analysis solutions were also performed using a 0.15 mL volume of $^{2}H_2O$, thorough rotamixing and then removal of the lower $^{2}H_2O$ phase prior to ^{1}H NMR analysis. ^{1}H NMR analysis of the above $^{2}H_2O$ micro-extraction solutions were performed on the JEOL-ECZ500 500 MHz spectrometer (University of Loughborough facility) operating at a frequency of 500.16 MHz. The acquisitional parameters were: zg30 pulse program with no dummy scans; acquisition time 3.166 s; 128 scans; receiver gain of 16; 131,072 datapoints; and, spectral width 41,377 Hz.

The spectra acquired on full $^{2}H_2O$ and $C^{2}H_3O^{2}H$ extracts of Products 1-4 (prepared as outlined in Section S2.2 above) were acquired on a Bruker AV-500 500 MHz spectrometer (University of Leicester facility, Leicester, UK) that was equipped with an Ultrashield plus 500 magnet and a 5-mm double resonance broad band (BBO) probe, and operating at a frequency of 500.13 MHz. The pulsing conditions were: zg30 pulse program with no dummy scans; 128 FIDs using 65,536 data points; an acquisition time of 3.17 s; and, a sweep width of 10,330 Hz.

Full details regarding the preprocessing of ^{1}H NMR profiles, the determinations of different aldehydic LOP classification, and their lower limits of detection and quantification (LLOD and LLOQ, respectively) are available in Section S2.4, below.

S2.4 Preprocessing of ^{1}H NMR spectral profiles: Determinations of classes of aldehydic LOPs in CLOs, and their lower limits of detection and quantification (LLOD and LLOQ respectively)

Aldehydic LOP regions of the spectral profiles acquired (*i.e.* those within the 5.40–10.20 ppm spectral range) were preprocessed by the application of a separate macro for the 'intelligent bucketing' processing sub-routine. These procedures were performed using the ACD/Labs Spectrus Processor 2018 software package (ACD/Labs, Toronto, Ontario, Canada M5C 1T4), and this generated a culinary oil dataset matrix that consisted of intelligently-selected buckets (ISBs) corresponding to the-C<u>H</u>O function signals of different aldehyde classes, *i.e. trans*-2-alkenals (*d*, δ = 9.49-9.51 ppm); *trans,trans*- and *cis,trans*-alka-2,4-dienals (*d*s, δ = 9.51-9.55 and 9.60-9.63 ppm respectively); 4,5-epoxy-*trans*-2-alkenals/acrolein (*d*, δ = 9.55-9.57 ppm); *n*-alkanals (*t*, δ = 9.74-9.76 ppm); 4-oxo-*n*-alkanals (*t*, 9.76-9.78 ppm); and, low-molecular-mass *n*-alkanals, *i.e.* ethanal, *n*-propanal and *n*-butanal (*t*, 9.79-9.80 ppm). A series of further unassigned aldehyde-C<u>H</u>O function signals were also notable within the δ = 9.40-10.20 ppm region (Figure 1), but two quantifiable resonances were those located within the δ = 9.78-9.79 and 9.80-9.82 ppm ISB regions (both multiplets), and these were labelled U1 and U2, respectively. Prior to commencing this intelligent bucketing process, all of the spectra were visually examined for any inherent distortions and manually corrected, if required. The electronic intensities of resonances corresponding to each of the above -C<u>H</u>O resonance ISBs were normalised to that of one encompassing all acylglycerol-C$\underline{H}_3$ function signals (δ = 0.82-0.99 ppm), so that their concentrations in each CLO sample could be expressed as μmol. or mmol. aldehyde per mol. of FA (μmol. or mmol./mol. FA). 'Between-TSE duration' sample coefficients of variation for all aldehyde class determinations made on the n = 3 replicated thermal stressing episodes varied from 3.8–12.7% for all oils investigated, whereas those for repeat determinations made on the same oil sample were ≤ 2.6%.

The lower limits of detection and quantification (LLOD and LLOQ, respectively) values for *trans*-2-octenal and *n*-hexanal (typical *trans*-2-alkenal and *n*-alkanal secondary LOPs) were computed from serial dilutions of them, along with a consideration of the 3σ and 10σ signal-to-noise approaches while using the *MNova* (Mestrelab, Santiago de Compostela, Spain) signal-to-noise ratio (SNR) software module. In neat C^2HCl_3 solution, LLOD and LLOQ estimates for *trans*-2-octenal were 10 and 33 μmol./L, respectively, whereas these values were 8 and 27 μmol./L, respectively, for *n*-hexanal.

However, for the analysis of aldehydic LOPs in oil samples, these LLOD and LLOQ values were somewhat greater, specifically 36 and 120 μmol/kg, respectively, for *trans*-2-alkenals (equivalent to 11 and 36 μmol./mol. FA, respectively); 40 and 133 μmol/kg for *trans,trans*-alka-2,4-dienals (equivalent to 12 and 40 μmol./mol. FA, respectively); and, 22 and 73 μmol/kg for *n*-alkanals (equivalent to 6 and 22 μmol./mol. FA, respectively) for the *ca.* 1/3 (v/v) diluted CLO samples prepared as described in section 2.3 above. These higher values are attributable to the influence of significant oil medium matrix effects at *ca.* 1/3 oil/C^2HCl_3 dilution ratios. Notwithstanding, these values improved *ca.* two-fold with lower C^2HCl_3 dilution levels of these culinary oils (*i.e. ca.* 2/3 rather than 1/3 dilutions), and also the acquisition of larger numbers of ^{1}H NMR scans (up to 1,024).

Calibration curves for typical *trans*-2-alkenals and *n*-alkanals (0-500 μmol/L^{-1} and 0.50–30.00 mmol./L) were linear, with R^2 values ≥ 0.995 for neat C^2HCl_3 solutions, and ≥ 0.984 for aldehyde-treated C^2HCl_3-diluted CLO samples prepared, as described above.

S3 Materials for Non-NMR Analyses

All of the standards were purchased from Millipore–Sigma, including the Folin-Ciocalteu reagent, and 2,4-dinitrophenylhydrazine. All other solvents and reagents were purchased from Fischer Scientific Ltd. (Loughborough, UK).

S4 Extraction Process for the Analysis of Total Phenolics, Flavonoids, Flavonones, Anthocyanins, Tannins, and Carotenoids, and Chlorophylls (Sections S5, S6, S7, S8, S9, and S11, respectively)

The CLO samples were added to 2:1 (v/v) chloroform:methanol, and then rotamixed to ensure dissolution. The solution was then allowed to equilibrate at ambient temperature for at least 1 hr.,

and then had a methanol-equivalent volume of doubly-distilled water added, and all of the samples were then thoroughly rotamixed. The samples were then centrifuged to separate the layers and the aqueous phase was collected for testing purposes.

S5 Total Phenolics

Total phenolics were determined via the spectrophotometric Folin-Ciocalteu assay [S3,S4]. These concentrations were estimated in triplicate by diluting 100 μL aliquots of the above (Section S4) aqueous phase that was collected with a 4.50 ml volume of doubly-distilled water and then adding 100 μl of a 2.0 mol./kg solution of the Folin–Ciocalteu reagent. Subsequently, 0.30 mL of 2.00 % (w/v) of a sodium carbonate solution in doubly-distilled water was added, and the samples were allowed to equilibrate at ambient temperature for 2.0 hr. Sample absorbances were monitored at 760 nm and then compared to those arising from gallic acid calibration standards at concentrations ranging from 15.6 to 500.0 μg mL [S3,S4].

S6 Flavonoids

Flavonoids and flavonones (Section S7 for the latter) were estimated by colourimetric methods involving aluminium chloride and 2,4-dinitrophenylhydrazine, respectively [S5].

The total flavonoids were determined in triplicate by diluting 125 μL of the above (Section S4) aqueous phase with 1.025 mL of water, and then adding 37 μL of 5% (w/v) sodium nitrite solution. These mixtures were then equilibrated at ambient temperature for a period of 5 min. Subsequently, 75 μL of 10% (w/v) aluminum chloride solution was added, and these solutions were again equilibrated at ambient temperature for 5 min. Finally, 250 μL of 4.0 mol//L sodium hydroxide was added with swirling to remove any precipitates. The sample absorbances were monitored at 510 nm, and then compared to those of standard curves generated from catechin calibration standard solutions that ranged from 7.8 to 250.0 μg/mL to determine their concentrations [S5].

S7 Flavonones

The total flavonones were determined in triplicate by adding 0.50 mL of the above (Section S4) aqueous phase to 1.00 mL of 1% (w/v) solution of 2,4-dinitrophenlyhydrazine in methanol. The solutions were then rotamixed and equilibrated at 50ºC for 50 min. Subsequently, 2.50 ml of a 1.00% (w/v) solution of potassium hydroxide in ethanol was added to each sample tube, followed by rotamixing. These samples were equilibrated at ambient temperature for 15 min., and were then clarified via centrifugation. Sample absorbances were monitored at 512 nm and then compared to those of standard naringin calibration standards that ranged from 62.5 μg to 2.0 mg/mL to determine the total CLO flavanone concentrations [S5].

S8 Anthocyanins

The total anthoycanins were determined in triplicate by a pH differential method [S5]. The samples had two separate 1.00 mL portions of the above (Section S4) aqueous extract dispensed per replicate, and had their pH values adjusted to 1.0 or 4.5 with hydrochloric acid or sodium acetate buffer, respectively, to a final volume of 2.00 ml. The absorbances were monitored at 520 and 700 nm, with the adjusted absorbance value representing that at 520 nm minus that at 700 nm. The adjusted absorbance measurement that was obtained at a pH value of 1.00 was then further adjusted by subtracting that obtained at pH 4.5, and then converted to cyanodin equivalents by multiplying by a molecular mass of 449.2 g/mol., and then dividing by a millimolar excitation value of 26.9 to yield mg anthocyanin/mL extract levels [S6].

S9 Tannins

The total tannin contents were determined by the acidified vanillin assay system [S4]. These levels were determined in triplicate by adding 0.25 ml volumes of the above aqueous phase (Section S4) to 1.00 mL of a 4% (w/v) solution of vanillin in methanol, followed by 0.50 mL of concentrated

hydrochloric acid; the samples were then equilibrated at ambient temperature for a period of 20 min. The sample absorbances were monitored at 500 nm, and total tannin levels were estimated from a catechin calibration standard curve that ranged from 15.6 to 500.0 μg/mL [S7].

S10 Vitamins A and E

A reversed-phase HPLC method assayed tocopherols (α- and γ-tocopherols) and retinol [S8]. A sample of 1.00 g of CLO was added to a 1.00 ml aliquot of methanol, and the methanol phase was then collected and partially dried. Aliquots (25 μL) of this dried methanol phase were injected on a Waters HPLC system with a 600S controller and pump unit, 717 plus autosampler, and a 2996 photodiode array detector. Separation was achieved with an ES Industries Sonoma C18(2) 5μ 100Å 25 cm x 4.6 mm column, while using a mobile phase of methanol:acetonitrile:tetrahydrofuran (75:20:5 (v/v/v)) at a flow-rate of 0.60 mL/min. Detection was at 325 nm for retinol, 290 nm for tocopherols, and 450 nm for carotenoids; the sample analyte peak areas were compared to those of calibration standards of α-, γ-, and δ-tocopherols, β-carotene, and retinol [S8].

S11 Carotenoids and Chlorophylls

Chlorophylls A and B were spectrophotometrically determined according to [S9,S10]; carotenoids (β-carotene and total) were also determined by a spectrophotometric assay [S10].

Absorbance values of the aqueous phase (Section S4) were monitored at 470, 653, and 666 nm [S10]. Total carotenoids and chlorophylls A and B were determined from the calculations that are given below [S10]:

μg/mL Chlorophyll A = (15.65 x A_{666}) – (7.34 x A_{653})

μg/mL Chlorophyll B = (27.05 x A_{653}) – (11.21 x A_{666})

μg/mL Carotenoids = (1,000 x A_{470}) – (2.86 x Chlorophyll A) – (129.2 x Chlorophyll B)/245

S12 Determination of ORAC Values

10 to 15 μg mass of oil was accurately weighed and then dispersed in 500 μl of 1:1 (v/v) acetone:water containing 7% (w/v) added methyl-β-cylcodextrin[8]. Each sample was then diluted 50-fold in 75 mmol./kg phosphate-buffered saline at pH 7.40, and then dispensed in triplicate as a 25 μL volume onto a 96-well plate. Each well was then treated with 150 μL of 0.30 nmol./L fluorescence solution, and their baseline fluorescence was monitored at 485 nm excitation and 520 nm emission. To commence the reaction, 25 μL of a solution containing 800 mg of 2,2′-azobis(2-methylpropionamidine) dihydrochloride/mL in 75.0 mM phosphate-buffered saline (PBS) at pH 7.4 was added to each well, and the fluorescence intensity was then monitored at 1.5 min. intervals for a total period of 2 hr. The overall florescence intensity over time was compared with that of 6-hydroxy-2,5,7,8-tetramethylchoman-2-carboxylic acid (Trolox) solutions that ranged in concentration from 0.80 to 100 μg/mL in methanol as the calibration standards [26,S11].

S13 Biogenic Amine Analyses

Biogenic amine analysis by the LC/MS/MS technique followed the method of MWL HPLC 033, which is based on research articles by *MDS Analytical Technologies* and *Applied BioSystems*. For the simultaneous analysis of a total of 11 of these agents, the fixed aliquots of CLO samples were filtered using a 0.45-μm filter paper, shaken with a 20 mL volume of 70% (v/v) methanol/30% (v/v) water for 20 min., and then centrifuged at 7,000 rpm at 4°C for a further period of 20 min. Clear supernatants were then transferred to 1.7 mL volume amber auto-sampler vials for LC/MS/MS analysis.

A Dionex UltiMate® 3000 Rapid Separation LC (RSLC) system consisting of a pump, vacuum degasser, auto-sampler, column compartment, and a variable wavelength electronic absorption detector system, and that had the ability to operate up to 800 bar, was employed in combination with an Applied Biosystems/MDS Sciex API 3200TM mass spectrometer.

Acetonitrile was added to the LC eluent at a rate of 0.40 mL/min. at the Turbo VTM source's "tee" piece in order to increase sensitivity, and this facilitated the generation of dry ions during the early part of time program, at which the water content was high. A 3-μm 50 x 2.1 mm Pinnacle® DB PFPP column was used for this analysis; this system was found to be optimal for biogenic amine separations, rapid analysis, and robustness. Mobile phase A consisted of water containing either 0.05 or 0.10% (w/v) of the ion-pair reagent trifluoroacetic acid (TFA), whereas mobile phase B was acetonitrile containing a matching concentration of TFA.

Analyte species were monitored in positive ion, multiple-reaction monitoring (MRM) mode for the MS/MS detection system.

S14 Total Protein, Collagen, and Ammonia analyses

S14.1 Total protein content

The total protein content analysis was conducted using the combustion method MWL FO 014, which is based on AOAC 992.15 and USDA/FSIS CLG-PRO04.03 [S12]. The standard minimum reporting level was 0.1% (w/w)

S14.2 Collagen detection and analysis: Product 4

Collagen was determined by (1) sodium dodecyl sulfate polyacrylamide gel electrophoresis (SDS-PAGE) and (2) assay of total hydroxyproline following protein hydrolysis using the HPLC method.

S14.2.1 SDS-PAGE analysis

S14.2.1.1 Acetone precipitation of proteins

Aliquots (0.40 mL) of cold (-20°C) acetone were transferred to a microcentrifuge tube, and to these were added 0.10 mL volumes of CLO samples. The mixtures were then vortex-mixed and equilibrated at -20°C for a period of 60 min. Mixtures were centrifuged (10 min. at 13,000 x g) and the supernatant discarded. The acetone was then allowed to evaporate for a period of 30 min.

S14.2.1.2 Collagen extraction process

Subsequently, collagenous species were extracted from the pellet medium with two separate methods: acid-and pepsin-soluble collagens (ASC and PSC, respectively).

For the ASC protocol, the pellets were treated with sodium hydroxide, 10% (v/v) butyl alcohol and 0.50 mol./L acetic acid, and the mixtures were then left to equilibrate for a period of 72 hr. at ambient temperature. The samples were then centrifuged at 20,000 x g for 1.0 hr., and the solute precipitated with added NaCl.

The PSC extraction method also involved the prior addition of sodium hydroxide, 10% (v/v) butyl alcohol, and 0.50 mol./L acetic acid solution to samples, but this was followed by treatment with a 10% (w/v) pepsin medium. These mixtures were then centrifuged (20,000 x g) for 1.0 hr., the solute precipitated with added NaCl, and the sample was again centrifuged (20,000 x g) for a 1.0 hr.

S14.2.1.3 SDS-PAGE analysis

A 50 μL volume of sample solution was pre-treated with 10 μL of a 1% (w/v) pepsin medium, and the samples were left to equilibrate at ambient temperature for a period of 20 min. The aliquots (45 μL) were then mixed with 15 μL of 4X LDS sample buffer, samples were equilibrated at 65°C for 15 min., and then cooled to ambient temperature. The SDS-PAGE apparatus was assembled and a pre-cast gel was employed. The chamber was filled with 1X MOPS running buffer; 10 μL of a protein ladder was loaded into the first well, and 20 μL of collagen standards, reference and test samples were loaded into the remaining gel wells. Subsequently, the gel was run at 200 V and 65 mA for *ca.* 50 min. Following the run, the proteins in the gel were fixed with fixing solution. The gel was stained with Coomassie blue staining solution, and then de-stained with the de-staining solution. A typical camera device then captured the gel image.

S14.2.1.4 Gel readings and interpretation

Collagen standards: On the gels, three typical bands within the high-molecular-mass area (located on the top area of the gel), i.e. single bands for the α- and β-chains (2 α's for the latter), and one for the γ-chain (3 α's) were observed.

Hydrolysed collagen: Hydrolysis into smaller fragments gave rise to the appearance of many bands in the low-molecular-mass area that was located at the bottom of the gel. However, when the concentration of hydrolysed collagenous derivatives was high, these appeared as a 'smear' on the gel.

Extracts of pepsin-solubilised collagen (PSC method) gave rise to a pepsin protein band on the gel that was within the 30-40 kDa range.

S14.2.2 Analysis of collagen in Product 4 as hydroxyproline equivalents by HPLC

Product 4 CLO collagen was hydrolyzed to its constituent amino acids. The amino acids were then derivatized for improved HPLC separation purposes.

Primarily, collagen was separated from the fish oil by extraction with both water and hexane; the collagen was dissolved or suspended in the aqueous phase. A 1.00 mL volume of this aqueous layer was then treated with 6.0 mol./L hydrochloric acid in a sample tube, which was then sealed and the mixture was heated overnight. Subsequently, the reaction mixture was cooled and neutralized to a pH value with the 7–8 range with aqueous sodium hydroxide. The volume of the mixture was then increased to 5.0 mL with water.

The tube was tight-sealed and the mixture heated at 78°C for a period of 2.0 hr. after adding 1.00 mL of potassium phosphate buffer and an equivalent volume of a phenylisothiocyanate (PITC) solution (Edman's reagent) in methanol (individual amino acid standards were also treated in the same manner). Following this derivatization reaction, solutions were centrifuged (or filtered) to remove any particulate matter, and then transferred to vials for analysis. The PITC derivatization reagent allowed for the use of a single wavelength to detect and quantitate individual amino acids.

The amino acids were separated on a Thermo-Fisher Betasil C18, 5 μm, 4.6 x 250 mm column, while using a 0.05% (w/v) phosphoric acid solution in water as mobile phase A, a 0.05% (w/v) phosphoric acid solution in methanol as mobile phase B, a flow-rate of 1.00 mL/min. and a PDA detection system pre-set at a monitoring wavelength of 254 nm.

S14.3 Ammonia analysis of Product 4

Ammonia in Product 4 was determined as ammoniacal nitrogen using the SM 4500-NH3 C-(1997) method, which involves a preliminary distillation step followed by an acid-base titration step.

S15 Moisture Contents

AOCS Official Method Ca 2a-45 determined the water contents of different batches of Product 4 [S13]. This method involved a distillation process that was conducted with an immiscible solvent, and it might be applied to a range of fats and oils, including emulsions.

S16. Discussion of the Relatively Minor Contributions of Low-Molecular-Mass Phenolics, Flavonones, Biogenic Amines and Polyphenolic Tannins Towards Product 4's Superior Protective ORAC Indices

Contributions of low-molecular-mass phenolics, flavonones, biogenic amines, and polyphenolic tannins towards Product 4's very high protective ORAC values, albeit relatively minor ones, are explicable by the availability of peroxidatively-significant levels of a complex pattern of these agents therein, and further aromatic compounds were directly ^{1}H NMR-observable in *ca.* 1:2 (v/v) CLO:C^2HCl_3-diluted solutions. Moreover, the identification of a range of coupled ^{1}H NMR-detectable aromatic resonances in both the ^2H_2O- and $C^2H_3O^2H$ extracts of Product 4 confirmed the assignments of side-chain amine function-containing aromatic/phenolic compounds,

particularly 2-phenylethylamine and tyramine, which were also directly ^{1}H NMR-observable in C^2HCl_3 solution. Indeed, the 2-phenylethylamine and tyramine levels that were measured by an alternative technique (LC/MS/MS) were found to range from 0.14–0.71 and 0.06–0.64 mmol./kg, respectively, in this CLO, and, hence, are readily ^{1}H NMR-detectable and -quantifiable. At least some of these classes of antioxidants detected may be considered as multifunctional in nature, as discussed further below. All of the primary and secondary amine species detected in Product 4 were absent from all other CLO products investigated here, as expected in view of their non-fermented nature.

For experiments involving investigations of the effects of exposing Product 4 to a 90 min. TSE on its biogenic amine concentrations, the above resonances that were assigned to 2-phenylethylamine and tyramine were completely removed from its ^{1}H NMR profiles following this heating duration. Indeed, these signals were eliminated from such spectra at TSE heating periods of ≥ 10-20 min. Moreover, the typical results that were acquired from the HPLC method also employed for biogenic amine analysis (section S13) were 2-phenylethylamine (0.61 mmol./kg), tyramine (0.49 mmol./kg) tryptamine (0.11 mmol./kg), and total primary and secondary biogenic amine functions (1.32 mmol./kg) prior to, and none detectable whatsoever in n = 3 replicate samples following the 90 min. heating phase. These data clearly indicate the oxidative consumption of three of the major biogenic amines (2-phenylethylamine, tyramine, and tryptamine) when Product 4 is subjected to TORA TSEs, presumably via their impairment of the thermally-induced peroxidation of O-3 FAs and MUFAs in this fermented CLO product, and/or their direct chemical reactions with secondary aldehydic LOPs generated therefrom. However, it is also possible that such amines may be partially thermally-degraded at the high TSE temperature employed, or that there are some evaporative losses of these when exposed to such temperatures. The b.pts of 2-phenylethylamine, tyramine, and tryptamine are all above the 180°C temperature employed in our TSE protocol (198, 206, and 379°C, respectively). Therefore, significant amounts of the first two of these biogenic amines, which have b.pts only *ca.* 20°C higher than the temperature employed for these experiments, may be volatilised during their course.

The antioxidant actions of the phenolic biogenic amine tyramine that are found in Product 4 (mean concentration 0.36 mmol./L) may be rationalized by its chain-breaking antioxidant effects, and this may offer contributions towards the potent resistance of UFAs, particularly O-3 FAs, present in this fermented CLO to thermally-induced oxidative damage observed herein. Moreover, the other major biogenic amine present, 2-phenylethylamine, which was present at higher levels (mean 0.55 mmol./L), might also exert some significant antioxidant effects, and the complete consumption of these biomolecules during exposure to TSEs for a period of 90 min. (Section 3.1) is consistent with these proposed activities. Stevenato *et. al.* [S14] provide further evidence for the antioxidant characteristics and capacities of these biogenic amines, finding that in view of its phenolic structure, tyramine (along with serotonin, L-norepinephrine, (-)-epinephrine, and dopamine) exerted powerful lipid peroxyl radical (LOO•)-scavenging activities, whereas the antioxidant actions of the aliphatic polyamines spermine, spermidine, putrescine, cadaverine, histamine, and tryptamine, which were also monitored in the study, were ascribable to their abilities to act directly on the O_2-consuming species featured in the PUFA peroxidation process, *i.e.* neutralisation of linoleic acid-derived carbon-centred pentadienyl radical moieties involved, presumably via amine function hydrogen atom (H•) donation. Indeed, this investigation also found that linear aliphatic polyamine species, *e.g.* spermine, spermidine, putrescine, and cadaverine, did not exert any activity when tested in a 2,2-diphenyl-1-picrylhydrazyl (DPPH) radical scavenging assay, or in an enzymatic method for evaluating such antioxidant capacities, whereas amines also containing phenolic functional groups were very effective when evaluated in both of these systems. However, all of the amines tested, both aliphatic and aromatic, were able to suppress O_2 consumption by peroxidising linoleic acid present in sodium dodecyl sulphate micelles, the above aliphatic ones responding to this almost as effectively as tyramine. Moreover, indirect laboratory measurements provided indications regarding the scavenging activity of polyamines towards PUFA-sourced alkyl or peroxyl radicals [S15].

The consumption of phenolic antioxidants during TSEs, as observed here for tyramine (section 3.1), is consistent with the effects previously described for α-TOH and synthetic dietary antioxidants, such as butylated hydroxytoluene (BHT), in recycling UFA peroxidation processes taking place during high temperature frying practices [S16]. Indeed, Gómez-Alonso *et. al.* [S17] reported that the total antioxidant activities of phenolic extracts of virgin olive oil (monitored as ORAC values) decreased at a rapid rate during 6 x 10 min. duration frying processes at 180°C, *i.e.* from a total activity of > 0.74 being observed prior to heating to a value < 0.25 mmol. trolox equivalents/kg following the final heating stage.

Although the α-TOH detectable in Product 4 might also provide a contribution towards its very high ORAC values, its peroxyl radical-trapping actions are expected to be very weak in view of its very low levels found therein (0.03–0.19 mmol./kg). Furthermore, the concentrations of carotenoids measured in this product (total levels 2.2-5.3 mg/L), including β-carotene (only 0.2–3.5 μmol./kg) are clearly insufficient for combating the thermally-induced oxidation of its O-3 FA constituents, *i.e.* are unable to exert any significant chain-breaking antioxidant effects under our experimental conditions. Notably, α-TOH is also susceptible to heat-induced degradation at temperatures that are relevant to those of standard frying practices [S18], as indeed are carotenoids [S19]. A further investigation [S20] has also reported the decomposition of α-TOH during high-temperature frying episodes and, in our study, the complete consumption of tyramine and other amines observed might also be ascribable to their thermal instabilities, in addition to their antioxidant and aldehyde-consuming actions.

Marty and Berset [S21] found that exposure of an authentic sample of all-*trans*-β-carotene to heating at 180°C for 2.0 hr. gave rise to the generation of oxidation products, in addition to various *cis*-isomers, as monitored by the HPLC method. They also demonstrated that increases in the air circulation level enhanced its degradation via the promotion of its O_2-mediated oxidation. Moreover, the b.pts of α-TOH and β-carotene are 200–220 and 178–179°C, respectively [S22], and, hence, there are expected to be significant evaporative losses of both these antioxidants at a temperature of 180°C, particularly for the latter.

Further phenolics that are conceivably present in fermented fish oil products include bromophenols, which again have the capacity to terminate the autocatalytic lipid peroxidation sequence. Notwithstanding, the concentrations of these agents that are putatively present in Product 4 are most likely insufficient for them to act in a chain-breaking antioxidant capacity. Indeed, maximal total bromophenol contents that were found in fish muscle and stomach were found to be only *ca.* 350 and 600 μg/kg, respectively, in a previous investigation [S23].

S17 ^{1}H NMR Detection of Aromatic Biogenic Amines in Deuterium Oxide and Deuteromethanol Extracts of CLO Products

Further exploration of the sharp antioxidant/amine signals that were found in the ^{1}H NMR profiles of C^2HCl_3 solutions of Product 4 was performed by the ^{1}H NMR analysis of ^2H_2O and d_4-deuteromethanol ($C^2H_3O^2H$) extracts of this CLO, the former of which contained clear resonances that were assignable to aromatic compounds, including 2-phenylethylamine (Figure S3). Indeed, aromatic resonances that were present in reference ^{1}H NMR spectra of this biogenic amine in ^2H_2O at pH 7.4 [32] were also featured in the spectra of these aqueous CLO extracts (*i.e.* δ = 7.34 and 7.40 ppm), and this served to confirm its identity. However, the intensity of the 7.34 ppm signal was affected by a small degree of overlap with a less intense one. Moreover, weaker intensity resonances were also visible in spectra that were acquired on these extracts, and two doublets located at δ = 6.89 and 7.20 ppm are assignable to tyramine's aromatic protons [32]. In contrast, spectra of corresponding ^2H_2O extracts of Products 1-3 were found not to contain any aromatic resonances whatsoever, although significant levels of the MDA degradation product formate [40] (presumably as formic acid in C^2HCl_3 solution), were detectable in Product 2.

In addition to ^{1}H NMR resonances arising from further fermentation products, *e.g.* the organic acids acetate, lactate, and propionate (presumably present as their carboxylic acid adducts in the Product 4 oil medium), and the terminal triacylglycerol hydrolysis products glycerol and free FAs,

a number of broad resonances (predominantly aliphatic) were also found in these ^2H_2O extract spectra, and these are likely to arise from collagen and/or lower molecular mass collagen degradation products that are derived from the Product 4 CLO medium (data not shown). Also notable was an increased line-width at half-height of the low-molecular-mass fermentation product resonances, for example, a propionate-CH_3 function signal at δ = 1.05 ppm, which had a $\Delta v_{1/2}$ value of 2.9 Hz. This provided evidence for its binding to collagen polypeptides; from our biomolecular ^1H NMR reference data that were acquired on aqueous solutions of propionate in the absence of macromolecules or alternative line-broadening agents; this value was found to be ≤ 1.5 Hz.

Corresponding $C^2H_3O^2H$ extracts of Product 4 yielded ^1H NMR spectra that contained relatively intense coupled aromatic multiplet resonances centred at δ = 7.23 and 7.31 ppm, which again correspond to the aromatic resonances of 2-phenylethylamine (data not shown). Moreover, these spectra also contained two sets of intensity-correlated doublet resonances that were located at δ = 6.67/6.98 and 6.77/7.05 (data not shown), which are conceivably ascribable to phenol derivatives with a second aromatic substituent in the 4-position (*i.e. p*-substituted phenolic rings). Although no reference ^1H NMR spectral profiles are available for tyramine in this $C^2H_3O^2H$ solvent system, those of N-*cis*- and N-*trans*-feruloyl-tyramine derivatives exhibit coupled doublet resonances that are located at δ = 6.68/7.00 and 6.70/7.03 ppm, respectively [33], and, therefore, those observed in these Product 4 extracts are presumably assignable to tyramine and/or either one or more of its isomeric N-substituted derivatives. Alternatively, one set of these coupled signals might arise from the phenolic ring protons of its O-sulphate derivative [33]. Therefore, the results acquired from the ^1H NMR analysis of these extracts are consistent with those obtained from the ^2H_2O ones, albeit with a larger number of resonances visible; these data will be reported in detail elsewhere.

S18 Extractions of Proteins/Enzymes with Organic Solvents, and their Solubilities and Activities Therein

Notwithstanding, proteins do have limited solubility in organic solvents [S24], and Houen [S25] found that the solubility of bovine serum albumin and lysozyme in chloroform (containing 1% (v/v) ethanol) was 1.8 and 17.5 μmol./L. Intriguingly, a range of water-immiscible solvents, *e.g.* octane and ethyl acetate, have been shown to serve as outstanding media for many selective enzymatic reaction systems [S24].

The studies of Burlanek and Yousouf [S26] confirmed that nisin, a polycyclic peptide with bactericidal properties that are generated by *Lactobacillus lactis*, and that is also used as a food preservative, was readily extractable from *Lactobacillus leichmannii* (ATCC 4797) cultures into an interface between chloroform and these aqueous system-based cultures when employing this solvent as an extraction medium, as were further bacteriocins (pediocin PO2, bacillin 115C and subtilin) from alternative bacterial sources. These results may be consistent with those of our studies that are reported herein, and any collagenous proteins and/or peptides that are located within the CLO-fermented cod liver matrix interstitial layer may be incorporated into the upper oil medium in an emulsified or alternative form when it is removed from the solid fermenting phase; water contents of up to 1.0 % (w/w) found in this product may facilitate this process. However, Product 4 was commercially centrifuged prior to its isolation and bottling.

S19 Novel Antioxidant Properties of Usually Discarded Fish Components and Other Marine Products

From Nagatsuka's investigation [52], the head, bone, skin, and tail mixed parts of yellowtail and bream, scales of bream, and head of salmon, which have valuable nutritional values, are usually discarded in Japan and, in this study, these fish by-products were employed to prepare a 'Nikogori' gelatin gel. Furthermore, Nagatsuka's group found that chicken jelly collagen-derived 'Nikogori' soup exerted strong peroxyl and hydroxyl radical-scavenging capacities when assessed using chemiluminescence and electron spin resonance (ESR) methods [S27]. Indeed, lipids that are released from chicken wings into this soup medium form layers or emulsions, just as they may do

in the Product 4 CLO medium explored here, and the Nikogori samples are gelated by powerful peroxyl radical-scavenging collagen degradation peptide products that elute into it.

Moreover, Suetsuna *et. al.* [S28] documented the antioxidant activities of peptides that were derived from the peptic digestion of fish scale collagen, and concluded that acetic acid-extracted peptides from saury (*Cololabis saira*) collagen exerted the greatest effects in this context. Additionally, Han *et. al.* [S29] discovered that cod skin collagen-derived peptides that were obtained from industrial fishing by-products exerted protective effects against oxidative damage to Chang liver cells both *in vitro* and *in vivo*.

One very recent investigation [S30] found that four trypsin-digested, collagen-derived peptides from *Chondrosia reniformis* marine sponge exerted significant antioxidant activities in hydrogen peroxide (H_2O_2)- or quartz-stimulated macrophages, and also in appropriate cell-free systems (23–60% of reactive oxygen species (ROS)-scavenging ability); these peptides also functioned effectively in wound-healing tests.

Moreover, hydrolysates of abalone viscera, an industrial-derived waste product of abalone processing that arises from the actions of Alcalase protease, displayed an extremely high ORAC value, and it was also found to serve as a rich source of hydrophobic and antioxidant amino acids, both classes of which contributed to this peroxyl radical-trapping measure [S31]. These hydrophobic amino acids included cysteine, histidine, methionine, phenylalanine, tyrosine, and proline, collagen serving as a rich source of the latter. Indeed, these biomolecules have been shown to markedly increase the antioxidant potentials of peptides via their abilities to directly scavenge lipid-derived radical species, along with their electron- and proton-donating activities [S32].

Intriguingly, recently Zhao and Eun [S33] isolated ammonia-producing bacteria from n = 5 commercial fermented skate products and, out of a total of 88 strains of these species, seven were found to be hyper-ammonia producing bacterial isolates based on their abilities to generate this catabolite. Concentrations of ammonia, total volatile base, and trimethylamine nitrogen in these fermented skate samples were found to be as high as 4.86–7.31 g/kg (0.35–0.52 mol./kg), 3.3–7.0 g/kg (0.23–0.50 mol./kg), and 60-600 mg/kg (4.3–43.0 mmol./kg), and therefore it is not unexpected to find relatively high levels of this agent in the fermented CLO product that was explored in this study. Although ammoniacal nitrogen levels found in n = 2 typical samples of this product (mean 5.36 mmol./kg, equivalent to 75 mg/kg.) were confirmed by an alternative analytical method, detailed NMR evidence for the identification of this bacterial catabolite in Product 4 will be presented in a follow-up communication.

S20 Radical-Trapping Capacities of Ammonia in Foods

Although there is currently no information available in the scientific literature regarding the ability of ammonia to trap lipid-derived radical species that are featured in the autocatalytic peroxidation process, Balachanrana *et. al.* [S34] found that aqueous ammonia served as a very efficient hydroxyl radical (•OH) scavenger in chemical formulations that are irradiated with an acoustic field (*ca.* 1 MHz) at variable power densities. Indeed, the rate of •OH production in such media was found to be insignificant at power densities as high as 8 W/cm^2. Similarly, Huang *et. al.* [S35] concluded that the •OH radical generated from hydrogen peroxide (H_2O_2) photolysis sequentially oxidised NH_3 in aqueous solution to nitrite and nitrate anions (NO_2^- and NO_3^-, respectively), and NH_3 removal efficacies were influenced by its prior concentration and medium pH values. The mechanism of this process involved the prior abstraction of a H• atom from this substrate by •OH to yield •NH_2, a reaction that had a second-order rate constant of 1.0×10^8 $mol.^{-1}$ s^{-1}. Subsequently, •NHOH is formed.

Therefore, it is conceivable that ammonia detectable in Product 4 will serve to effectively scavenge lipid peroxyl (LOO•) and related radicals in Product 4 during thermally-stimulated peroxidation processes, although its putative presence as ion-paired NH_4^+ is likely to suppress this process. However, the total ammonia levels of *ca.* 6 mmol./kg indicate that it will remain a powerfully competitive scavenger in relation to the concentrations of phenolic and biogenic amine antioxidants that are present in this product.

The FDA has specified that the concentrations of ammonia and ammonium ion salts that are normally present in food products do not present a significant health risk to humans, although a number of restrictions have been placed on ammonium salt contents permitted in processed food products [S36]. For example, the maximum allowable levels in such foods are 0.04–3.2% (w/w) ammonium bicarbonate in grain, snack foods, reconstituted vegetables, and baked goods, and 2.0% (w/w) ammonium carbonate in gelatins, puddings, and baked goods. The content that was found in product 4 was equivalent to < 0.01% (w/w).

S21. Antioxidant Properties of Biogenic Amines

Of particular relevance to this investigation, a further study [S37] explored the antioxidant properties of polyamines in experimentally heated soyabean oil via the Rancimat® method set at a temperature of 110°C. This study found that both spermine and spermidine enhanced the oxidative stability of this culinary oil, with the former displaying greater potency in this context. Moreover, added concentrations of 0.10–0.60 g/kg of these polyamines, corresponding to 0.49-2.97 and 0.69-4.13 mmol./kg for spermine and spermidine, respectively, were more effective than butylated hydroxyanisole (BHA), butylated hydroxytoluene (BHT), tocopherol, and a rosemary extract when tested in this system, and also more effective than a green tea extract when added at levels of 1.96–2.97 and 2.76–4.13 mmol./kg, respectively. Therefore, these data provide evidence for the novel antioxidant actions of such polyamine species, which appear to be more powerful than those of traditional phenolic antioxidants, and also those from rich plant extract sources of such agents. These added amine concentrations are not markedly higher than those of 2-phenylethylamine and tyramine determined in Product 4 here (mean levels of 0.55±0.28 and 0.36±0.23 mmol./kg, respectively), although little or no spermine, or spermidine, were detectable therein (Table 2).

Therefore, in addition to collagen and its hydrolytic degradation products, and also possibly ammonia, the ORAC test evaluation results that were acquired on the Product 4 formulation also appear to be partially attributable to high levels of biogenic amines, such as 2-phenylethylamine and tyramine present in product 4, which act as antioxidants, either through the classical established chain-breaking mechanisms that are available for the latter, or via the abilities of their amine functions to directly neutralise carbon-centred pentadienyl and trimonoenyl radicals that are produced from the H• abstraction of PUFA and MUFA substrates, respectively, and which are the immediate precursors of lipid peroxyl radicals that arise from the interaction of these reactive carbon centres with O_2. However, these biogenic amines were present in Product 4 at levels much smaller than its total peroxyl radical-trapping (ORAC value) capacity, and they were undetectable in Products 1–3. Notwithstanding, flavonones and tannins also detectable in Product 4 may also exert such protective effects, albeit only minor ones. Assuming an average molecular mass of 300 for the flavonone species detectable, which were present at a mean level of 896 mg/kg in Product 4, the molar concentration of such antioxidants therein would be *ca.* 3 mmol./kg, which is conceivably sufficient for these to offer a significant level of protection against the thermally-induced peroxidation of UFAs, particularly O-3 FAs (especially if they contain > 1 phenolic-O$\underline{H}$ function per molecule). Many flavonones retain phenolic-O$\underline{H}$ functions, and would therefore be expected to offer some, albeit minor, contribution towards the chain-breaking antioxidant potential of Product 4. However, at a mean level of only 112 mg/kg, polyphenol-based polymeric tannins are present at low, sub-millimolar concentrations in Product 4, and therefore their antioxidant activities in this context are expected to be less effective, *i.e.*, a lower level of competitiveness for radical reactants that are involved in the lipid peroxidation process than those of this product's biogenic amines and flavonones.

Additionally, collagen cross-links such as dehydro-hydroxlysinonorleucine, which maintain the structure and flexibility of this protein, are known to be susceptible to chemical attack by secondary aldehydic LOPs, particularly MDA, which arise from the peroxidation of O-3 FAs [S38]. Therefore, intact Product 4 CLO collagen, and more specifically its hydrolytic degradation products therein, have the ability to directly react with such aldehydes and, hence, chemically consume them in the CLO medium, although the intact form of this biomolecule would be expected to markedly

denature and degrade when exposed to the high-temperature TSEs that are employed in the present study.

Supplementary Materials Section Tables

Table S1. 1H NMR-determined mean ± SEM molar % fatty acid contents of products 1-4, specifically total saturated fatty acids (SFAs), UFAs and O-3 FAs, together with those of DHA and EPA. A total of n = 4 repeat determinations were performed for each CLO product tested.

Product	Mean±SEM SFAs (molar %)	Mean±SEM UFAs (molar %)	Mean±SEM Total O-3 FAs (molar %)	Mean±SEM DHA (molar %)	Mean±SEM EPA (molar %)
1	19.4±0.13	80.6±0.13	21.5±0.23	10.4±0.10	9.7±0.23
2	18.0±0.10	82.0±0.10	25.8±0.02	12.3±0.01	12.0±0.07
3	18.1±0.08	81.8±0.08	25.3±0.12	12.3±0.01	12.2±0.08
4	16.6±0.06	83.4±0.06	27.0±0.15	9.3±0.01	15.5±0.02

Table S2. Statistical significance (*p* values) of *post-hoc* ANCOVA analysis of the 'between-CLO products' source of variation for the experimental model employed (Equation 2). All *p* values are those arising from Tukey's *post-hoc* test. All further *post-hoc* comparisons involving products 1, 2 and 3 featured in this study did not attain statistical significance. Abbreviations: ns, not significant.

Post-hoc Comparison	*t*-2-Alk	*t,t*-A-2,4-D	4,5-E-*t*-2-Alk	*c,t*-A-2,4-D	*n*-Alk	4-O-*n*-Alk	U1	LMM *n*-Alk	U2
**1 *vs.* 4**	4.89 x 10^{-2}	8.16 x 10^{-3}	3.60 x 10^{-2}	1.02 x 10^{-4}	ns	1.91 x 10^{-2}	2.47 x 10^{-3}	1.67 x 10^{-3}	1.22 x 10^{4}
**2 *vs.* 4**	2.00 x 10^{-3}	6.50 x 10^{-5}	2.58 x 10^{-4}	1.62 x 10^{-8}	2.35 x 10^{-2}	1.08 x 10^{-2}	1.76 x 10^{-5}	2.11 x 10^{-4}	1.15 x 10^{-6}
**3 *vs.* 4**	1.00 x 10^{-3}	2.40 x 10^{-4}	9.10 x 10^{-5}	< 10^{-9}	1.70 x 10^{-2}	2.40 x 10^{-4}	7.30 x 10^{-6}	1.10 x 10^{-5}	1.10 x 10^{-5}
**1 *vs.* 2**	ns	ns	ns	2.45 x 10^{-2}	ns	ns	ns	ns	ns

Supplementary materials section figures

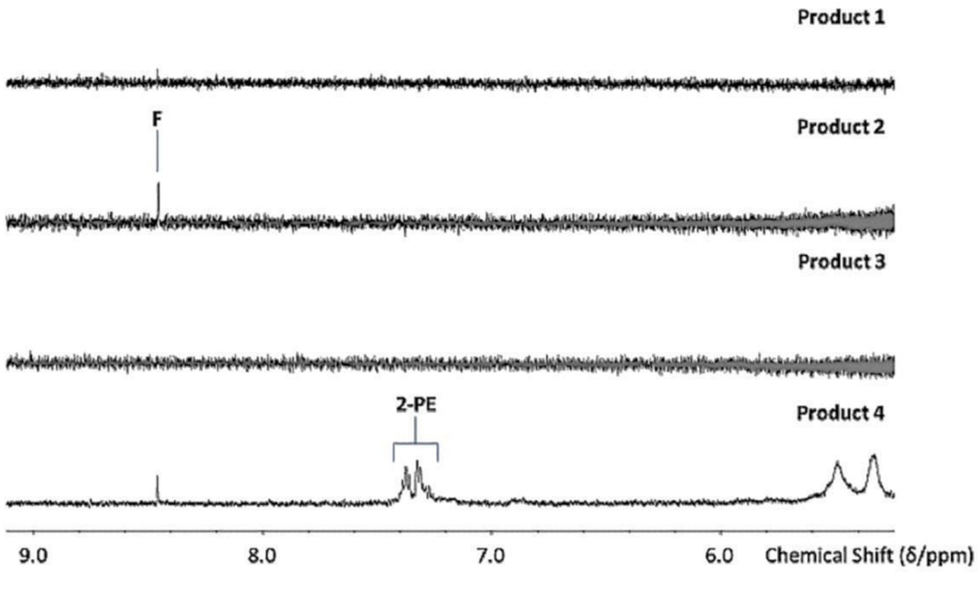

(a)

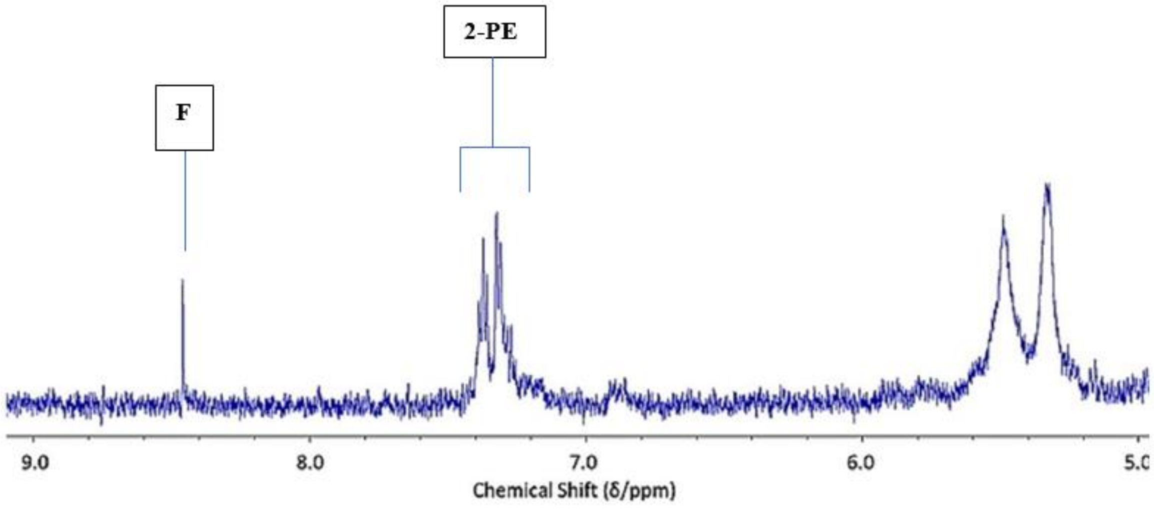

(b)

Figure S1. **(a)** Partial (4.90-9.10 ppm region of) the ^{1}H NMR spectra of $^{2}H_2O$ extracts of Products 1-4. **(b)** Expansion of this spectral region for Product 4. Abbreviations: F, formate-H; 2-PE, 2-phenylethylamine aromatic protons.

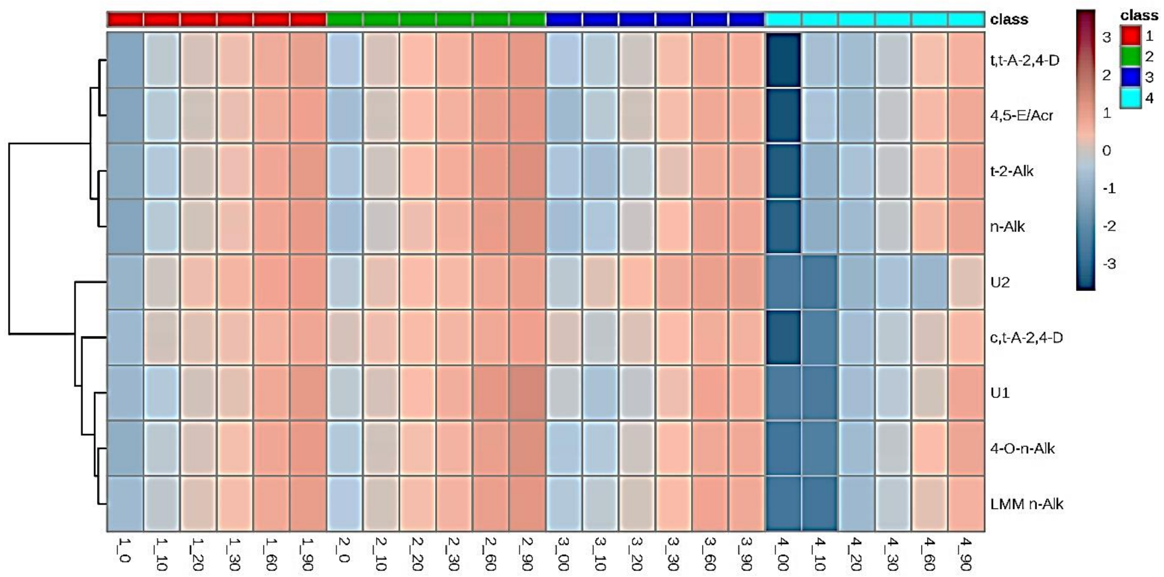

(a)

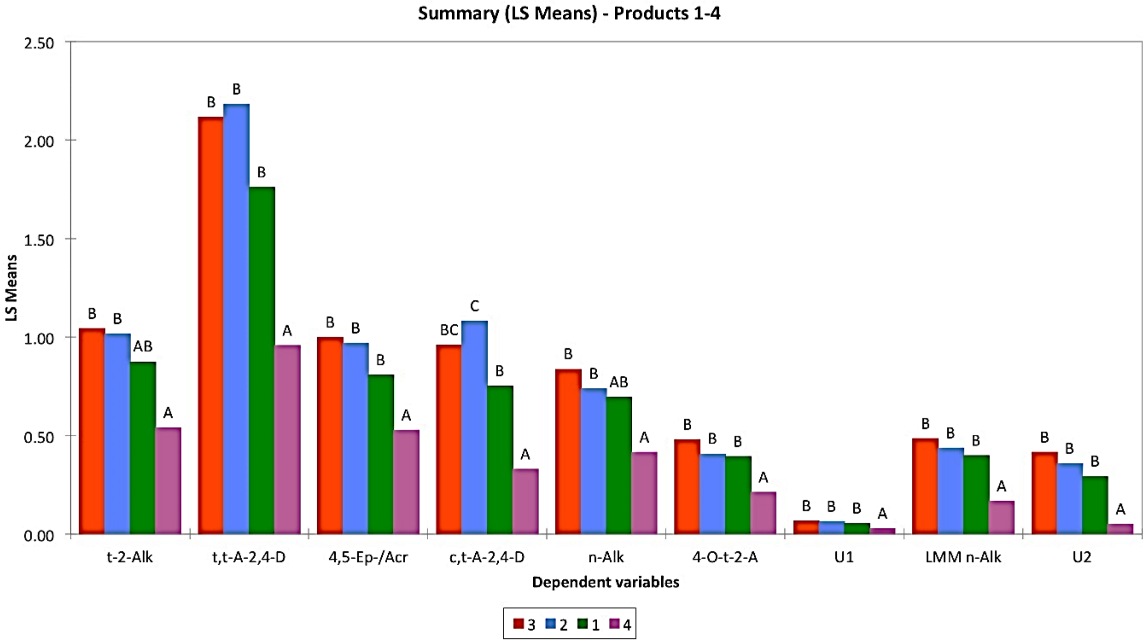

(b)

Figure S2. (a) Heatmap diagram showing the time-dependent generation of *trans*-2-alkenals (t-2-Alk); *trans*,*trans*-alka-2,4-dienals (t,t-A-2,4-D); combined 4,5-epoxy-*trans*-2-alkenals and acrolein (4,5-E-t-2-A/Acr); *cis,trans*-alka-2,4-dienals (c,t-A-2,4-D); *n*-alkanals (n-Alk); 4-oxo-*trans*-2-alkenals (4-O-E-t-2-A); low-molecular-mass *n*-alkanals (LMM n-Alk); Unidentified aldehyde classifications with resonances located within the δ = 9.78-9.79 and 9.80-9.82 ppm ISBs (U1 and U2 respectively) in CLO samples exposed to TSEs. Colour codes for Products 1-4 are shown in the top right-hand side panel. The abscissa axis depicts the CLO brands tested and the TSE heating time at 180°C (min.), for example 1_00 and 1_90 indicate Product 1 thermally-stressed for 0 and 90 min. respectively. Generalised log- (glog-) transformed aldehyde concentrations (mmol./mol. FA) are shown in the right-hand side y-axis: deep blue and red colourations represent extremes of low and high concentrations respectively. **(b)** Bar diagram plots of least squares mean (LSM) values of each aldehydic LOP classification for each of the 4 CLO products evaluated (colour codes for Products 1-4 are indicated on the abscissa axis; abbreviations for aldehyde classes correspond to those given in **(a)** above). A, B and C represent statistically significantly distinct products or groups of products, whereas AB and BC represent product LSM values which are intermediate between the two products/product groups indicated.

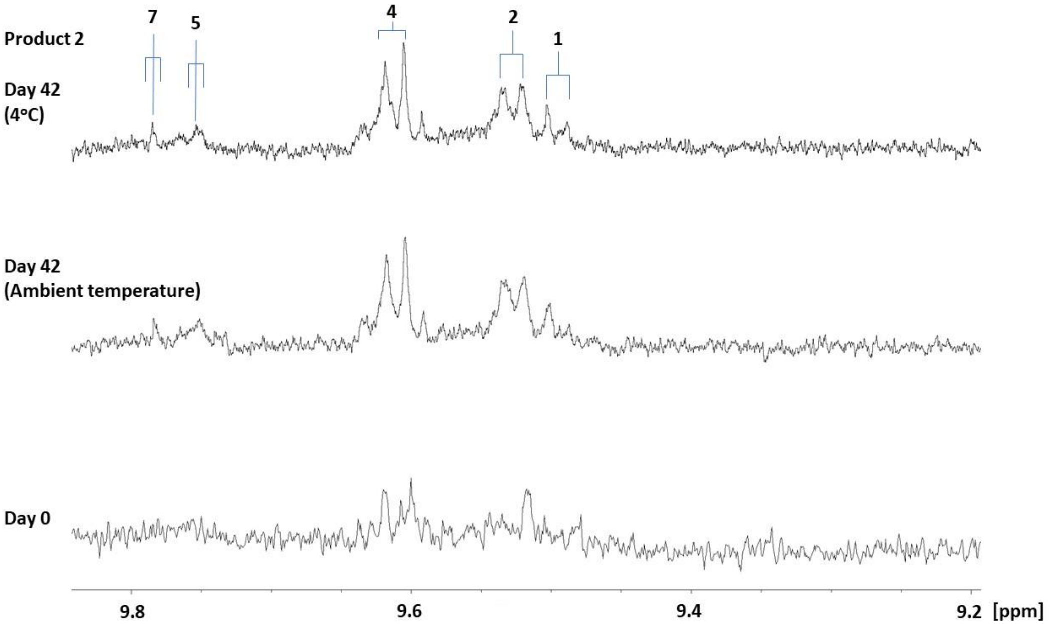

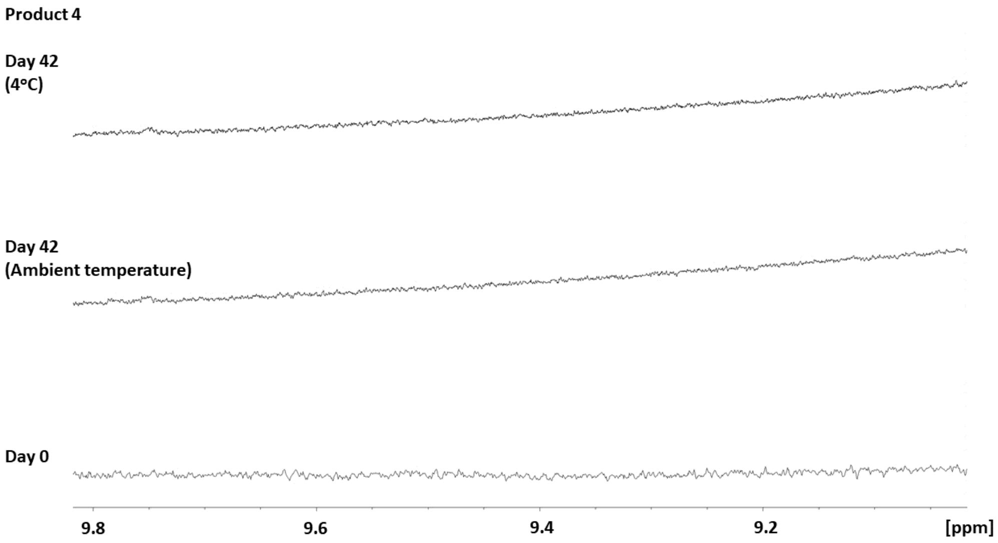

Figure S3. Aldehydic (9.0-9.8 ppm) proton regions of the 600 MHz ^{1}H NMR spectral profiles of Products 2 and 4 on day 0 (bottom), and after 42 days storage in the dark at ambient temperature (mean±SD 23±1°C) (middle) and in a darkened refrigerator at 4°C (top). Typical spectra are shown. Abbreviations: As Figure 1.

References

[S1] Jobling, M.; Leknes, O. Cod liver oil: Feed oil influences on fatty acid composition. *Aquacult. Int.* **2010**, 18(2), 223-230. DOI: 10.1007/s10499-008-9238-y

[S2] AOAC official method 996.06: Standard procedure for the determination of total, saturated, and unsaturated fat in foods using capillary GC-FID. **1996** (Revised **2001**).

[S3] Singleton, V.L.; Rossi Jr., J. A. Colorimetry of total phenolics with phosphomolybdic-phosphotungstic acid reagents. *Am. J. Enol. Vitic.* **1965**, 16, 144-158.

[S4] Adom, K.K.; Liu, R.H. Antioxidant activity of grains. *J. Agri. Food Chem.* **2002**, 50, 6182-6187.

[S5] Popova, M.; Bankova, V.; Butovska, D.; Petkov, V.; Nikolova-Damyanova, B.; Sabatini, A. G.; Marcazza, G. L.; Bogdanov, S. Validated methods for the quantification of biologically active constituents of poplar-type propolis. *Phytochem. Anal.* **2004**, 15(4), 235-240.

[S6] Shao, Y.; Xu, F.; Sun, X.; Bao, J.; Beta, T. Phenolic acids, anthocyanins, and antioxidant capacity in rice (*Oryza sativa* L.) grains at four stages of development after flowering. *Food Chem.* **2004**, 143, 90-96.

[S7] Bhat, R.; Sridhar, K.; Tomita-Yokotani, K. Effect of ionizing radiation on antinutritional features of velvet bean seeds (*Mucuna pruriens*). *Food Chem.* **2007**, 103, 860-866.

[S8] Talwar, D.; Ha, T.K.K.; Cooney, J.; Brownlee, C.; St J O'Reilly, D. A routine method for the simultaneous measurment of retinol, α-tocopherol and five carotenoids in human plasma by reverse phase HPLC. *Clinica Chimica Acta* **1998**, 270, 85-100.

[S9] Dere, S.; Gunes, T.; Sivaci, R. Spectrophotometric determination of chlorophyll - A, B and total carotenoid contents of some algae species using different solvents. *Tr. J. of Botany* **1998**, 22, 13-17.

[S10] Lichtenthaler, H.K.; Bushmann, B. Chlorophylls and carotenoids: measurement and characterization by UV-VIS spectroscopy. *Current Protocols in Food Analytical Chemistry* **2001**, p. F4.3.1-F4.3.8

[S11] Huang, D.; Ou, B.; Hampsch-Woodill, M.; Flangan, J.; Deemer, E. Development and validation of oxygen radical absorbance capacity assay for lipophilic antioxidants using randomly methylated β-cyclodextrin as the solubility enhancer. *J. Agric. Food Chem* **2002**, 50, 1815-1821.

[S12] King-Brink, M.; Sebranek, J. G. Combustion method for determination of crude protein in meat and meat products: collaborative study. *J AOAC Int.* **1993**, 76(4), 787–793.

[S13] AOCS Official Method Ca 2a-45 (Reapproved **2017**): Moisture in Fats and Oils, Distillation Method.

[S14] Stevanato, R.; Mariangela, B.; Fabris, S. In vitro preliminary evidences on the antioxidant properties of biogenic amines. *Pharmacol. Pharm.* **2013**, 4, 696-700. http://www.scirp.org/journal/pp) http://dx.doi.org/10.4236/pp.2013.49097

[S15] Fujisawa, S.; Kadoma, Y. Kinetic evaluation of polyamines as radical scavengers. *Anticancer Res.* **2005**, 25(2A), 965-970.

[S16] Brewer, M. S. Natural antioxidants: sources, compounds, mechanisms of action, and potential applications. *Comprehen. Rev. Food Sci. Food Safety*. **2011**, 10(4), 221-247.

[S17] Gómez-Alonso, S.; Fregapane, G.; Salvador, M. D.; Gordon, M. H. Changes in phenolic composition and antioxidant activity of virgin olive oil during frying. *J. Agricul. Food Chem.* **2003**, 51(3), 667-672.

[S18] Sabliov, C. M.; Fronczek, C.; Astete, C. E.; Khachaturyan, M.; Khachatryan, L.; Leonardi, C. Effects of temperature and UV light on degradation of α-tocopherol in free and dissolved form. *J. Am. Oil Chem. Soc.* **2009**, 86, 895–902. DOI 10.1007/s11746-009-1411-6

[S19] Caitlin, S.; Boon, D.; McClements, J.; Weiss, J.; Decker, E. A. Factors influencing the chemical stability of carotenoids in foods. *Crit. Rev. Food Sci. Nutr.* **2010**, 50(6), 515-532. DOI: 10.1080/10408390802565889.

[S20] Grootveld, M.; Atherton, M. D.; Sheerin, A.N.; Hawkes, J.; Blake, D. R.; Richens, T. E.; Silwood, C. J. L.; Lynch, E.; Claxson, A. W. D. In vivo absorption, metabolism, and urinary excretion of α,β-unsaturated aldehydes in experimental animals. Relevance to the development of cardiovascular diseases by the dietary ingestion of thermally stressed polyunsaturate-rich culinary oils. *J. Clin. Invest.* **1998**, 101(6), 1210-1218.

[S21] Marty, C.; Berset, C. Factors affecting the thermal degradation of all-*trans*-beta-carotene. *J. Agr. Food Chem.* **1990,** 38, 1063–1067.

[S22] ChemSpider Lab Network https://www.labnetwork.com/frontend-app/p/#!/moleculedetails/LN00196490

[S23] Oliveira, A. S.; Silva, V. M..; Veloso, M. C. C.; Santos, G. V.; De Andrade, J. B. Bromophenol concentrations in fish from Salvador, BA, Brazil. *Anais da Academia Brasileira de Ci frias* (*Ann. Brazil. Acad. Sci.*) **2009**, 81(2), 165-172. ISSN 0001-3765

[S24] Klibanov, A. M. Enzymatic catalysis in anhydrous organic solvents. *Trends Biochem. Soc.* **1989**, 15, 141-144. [S25] Houen, G. The solubility of proteins in organic solvents. *Acta Chemica Scandinavica* **1996,** 50(1), 68-70. DOI: 10.3891/acta.chem.scand.50-0068.

[S26] Burlanek, L. L.; Yousof, A.E. Solvent extraction of bacteriocins from liquid cultures. *Lett. Appl. Microbiol.* **2000**, 31, 193-197.

[S27] Nagatsuka, N.; Harada, K.; Ando, M.; Nagao, K. Measurement of the radical scavenging activity of chicken jelly soup, a part of the medicated diet, 'Yakuzen', made from gelatin gel food 'Nikogori', using chemiluminescence and electron spin resonance methods. *Int. J. Molec. Med.* **2006**, 18, 107-111.

[S28] Suetsuna, K.; Maekawa, K.; Chen, J-R.; Harada, K.; Hamada, M.; Shin, S-U. Separation and identification of antioxidative peptides from peptic digest of fish scale collagen. *J. Nat. Fish Univ.* (in Japanese) **2004**, 52: 57-62.

[S29] Han, Y.; Xie, J.; Gao, H.; Xia, Y.; Chen, A.; Wang, C. Hepatoprotective effect of collagen peptides from cod skin against liver oxidative damage in vitro and in vivo. *Cell Biochem. Biophys.* **2015**, 71, 1089. https://doi.org/10.1007/s12013-014-0313-x

[S30] Pozzolini, M.; Millo, E.; Oliveri, C.; Mirata, S.; Salis, A.; Damonte, G.; Arkel, M.; Scarfì, S. Elicited ROS scavenging activity, photoprotective, and wound-healing properties of collagen-derived peptides from the marine sponge *Chondrosia reniformis*. Mar. Drugs **2018,** 16**,** 465**.**

[S31] Jea, J-Y.; Park, S. Y.; Hwang, J-Y.; Ahn, C-B. Amino acid composition and in vitro antioxidant and cytoprotective activity of abalone viscera hydrolysate. *J. Funct. Foods* **2015**, 16, 94-103.

[S32] Samaranayaka, A. G. P.; Li-Chan, E. C. Y. Autolysis-assisted production of fish protein hydrolysates with antioxidant properties from Pacific hake (*Merluccius productus*). *Food Chem*. **2008**, 107(2), 768-776.

[S33] Zhao, C-C.; Eun, J-B. Isolation and identification of hyper-ammonia-producing bacteria from commercial fermented skates (Raja kenojei) *J. Food Sci. Technol.* **2018**, 55(12), 5082–5090. https://doi.org/10.1007/s13197-018-3447-9

[S34] Balachandrana, R.; Zhaob, M.; Dongb, B.; Brown, I.; Raghavana, S.; Keswani, M. Role of ammonia and carbonates in scavenging hydroxyl radicals generated during megasonic irradiation of wafer cleaning solutions. *Microelec. Eng.* **2014**, 130, 82-86. http://dx.doi.org/10.1016/j.mee.2014.10.022

[S35] Huang, L.; Li, L.; Dong, W.; Liu, Y.; Hou, H. Removal of ammonia by OH radical in aqueous phase. *Environ. Sci. Technol.* **2008**, 42(21), 8070-8075.

[S36] Agency for Toxic Substances and Disease Registry (ATSDR). **2004**. Toxicological profile for Ammonia. Atlanta, GA: U.S. Department of Health and Human Services, Public Health Service.

[S37] Mendonca, A. C. Atividade an antioxidante de poliaminas e comparacao com produtos naturais e sinteticos. PhD Thesis **2009**, Faculdade de Farmácia da University de Federal de Minas Gerais (UFMG), Belo Horizonte, MG, Brasil.

[S38] Slatter, D. A.; Paul, R. G.; Murray, M.; Bailey, A. J. Reactions of lipid-derived malondialdehyde with collagen. *J. Biol. Chem*. **1999**, 274(28), 19661–19669.
